# ZPY-CpG-Ch, a broad coverage vaccine against *Streptococcus pneumoniae*

**DOI:** 10.1126/sciadv.aee7981

**Published:** 2026-08-07

**Authors:** Teerawit Audshasai, Stavros Panagiotou, Lisa Crossman, Rong Xu, Lynn Verheijen, Chrispin Chaguza, Marie O’Brien, Aras Kadioglu

**Affiliations:** ^1^ Department of Clinical Infection Microbiology & Immunology, Institute of Infection, Veterinary & Ecological Sciences, University of Liverpool, The Ronald Ross Building, 8 West Derby St., Liverpool L69 7BE, UK.; ^2^ Department of Microbiology, Faculty of Pharmacy, Mahidol University, 447, Thanon Si Ayutthaya, Khwaeng Thung Phaya Thai, Khet Ratchathewi, Bangkok 10400, Thailand.; ^3^ SequenceAnalysis.co.uk, Norwich Research Park, Norwich NR4 7UG, UK.; ^4^ School of Biological Sciences, University of East Anglia, Norwich Research Park, Norwich NR4 7TJ, UK.; ^5^ Host-Microbe Interactions Dept. at St Jude Children’s Research Hospital, Danny Thomas Place, Memphis, TN 38105, USA.

## Abstract

Existing licensed pneumococcal vaccines, including Prevnar-13 and its higher-valency successors, have played a major role in reducing invasive pneumococcal disease worldwide. However, their effectiveness is increasingly threatened by the rise of multidrug-resistant strains and the shift in circulating serotypes toward those not targeted by current vaccines—a trend known as serotype replacement. These evolving challenges underscore the urgent need for next-generation vaccines with broader, serotype-independent protection—long considered the holy grail of pneumococcal vaccine development. Here, we report the discovery and preclinical evaluation of ZPY-CpG-Ch, a broad-coverage protein-based pneumococcal vaccine formulation. We assessed the protective efficacy and immunogenicity of ZPY-CpG-Ch in translational murine models, using both adult and neonatal mice, and tested various adjuvants, protein combinations, and doses, followed by challenge experiments with both vaccine- and non–vaccine-covered serotypes. Prevnar-13 was included as a benchmark. While Prevnar-13^®^ demonstrated robust efficacy, our results show that ZPY-CpG-Ch achieves greater cross-serotype protection. We attribute this enhanced efficacy primarily to T_H_17 (T helper 7)–biased immune responses. Together, these findings highlight the promise of genome-guided, protein-based vaccines in overcoming the limitations of current serotype-based approaches and represent a meaningful step toward a truly universal pneumococcal vaccine.

## Teaser

ZPY-CpG-Ch vaccine induces cross-serotype protection surpassing PCV13, offering a path toward universal pneumococcal vaccination.

## INTRODUCTION

*Streptococcus pneumoniae* remains a major global pathogen responsible for both respiratory and invasive infections, including pneumonia, meningitis, and sepsis. The burden of pneumococcal disease is particularly pronounced in young children and older adults, with global estimates of ∼1 to 1.5 million deaths annually (*1*). The European Centre for Disease Prevention and Control recognizes invasive pneumococcal disease (IPD) as a notable contributor to communicable disease morbidity and mortality across Europe and globally (*2*) (European Centre for Disease Prevention and Control, 2022). Clinical studies consistently report high case-fatality rates associated with pneumococcal pneumonia, with in-hospital mortality often ranging from 10 to 15%, even in high-income health care settings (*3*). Despite the availability of antibiotics, the clinical management of pneumococcal disease is increasingly complicated by the emergence of antimicrobial resistance. Extensive antibiotic misuse and the bacterium’s natural competence for horizontal gene transfer have facilitated the spread of resistance determinants among pneumococcal strains (*4*, *5*). As a result, World Health Organization has classified *S. pneumoniae* as a “priority pathogen,” highlighting its contribution to the global antimicrobial resistance crisis and the urgent need for new therapeutic options. In many settings—particularly those with limited health care infrastructure—treatment failure remains a persistent problem, underscoring the inadequacy of antibiotic therapy alone in mitigating disease burden. Consequently, effective vaccination strategies are essential for long-term control and prevention.

Vaccines have undeniably improved the prevention of pneumococcal disease. The 23-valent polysaccharide vaccine has been licensed for use in adults since 1983; however, as it induces a T cell–independent immune response, it does not elicit immunological memory. In contrast, pneumococcal conjugate vaccines (PCVs) drive T cell–dependent responses that generate high-affinity immunoglobulin G (IgG) and memory B cells. As a result, PCVs are highly immunogenic in infants and young children, and they not only protect vaccinated individuals but also reduce nasopharyngeal carriage of vaccine serotypes (*6*). The herd immunity effect further lowers transmission and disease even among unvaccinated people. For example, after the 7-valent PCV (PCV7) was introduced, many countries saw a ∼65% median reduction in vaccine-type IPD across all ages (*7*–*12*). Expanding to Prevnar-13 (PCV13) produced further declines in disease from its additional serotypes. In summary, routine PCV programs in children have undoubtedly led to lower incidence in IPD and non-IPD among both children and adults (*13*). Building on this, newer conjugate vaccines with broader serotype coverage have been licensed. In mid-2021, the US Food and Drug Administration approved two next-generation PCVs for adults: Merck’s 15-valent vaccine Vaxneuvance (PCV15) and Pfizer’s 20-valent vaccine Prevnar-20 (PCV20). Vaxneuvance contains the 13 serotypes from PCV13 and serotypes 22F and 33F, while Prevnar-20 includes the 13 PCV13 serotypes and 7 additional types (8, 10A, 11A, 12F, 15B, 22F, and 33F). These expanded-valency vaccines target serotypes that have become prominent causes of disease following earlier vaccine introductions. Most recently, in June 2024, the US Food and Drug Administration approved Merck’s 21-valent PCV (PCV21, branded Capvaxive) for use in adults aged 18 years and older. Capvaxive contains 21 serotypes—including 8 that are unique compared with Pfizer’s PCV20—and thus covers a broader spectrum of now circulating pneumococcal strains. According to US Centers for Disease Control and Prevention surveillance data, these 21 serotypes are responsible for ∼85% of IPD in US adults aged 50 years and older. In clinical studies, Capvaxive elicited robust immune responses across all 21 included serotypes, meeting or exceeding responses seen with PCV20 for the shared serotypes. The approval represents a major milestone in extending pneumococcal vaccine coverage to address evolving serotype epidemiology.

Despite this recent progress, however, the diversity of pneumococcal serotypes continues to pose a major challenge. There are now 101 known immunologically distinct serotypes (*14*, *15*), and any given serotype-dependent vaccine can only target a limited number of them. For example, use of PCV7 and PCV13 led to declines in vaccine-type IPD but concurrent rises in non–vaccine-type IPD. In children, the net effect remained strongly positive; however, in adults, the benefit can be offset if replacement serotypes circulate widely (*13*). This shifting landscape means that even PCV21—although highly protective—does not cover all serotypes in current circulation. The difficulty of covering many serotypes immunologically is inherent: Each added polysaccharide antigen can compete in the immune response, and manufacturing complex multivalent conjugates is challenging and expensive. Many scientific commentaries have emphasized that broad-spectrum or serotype-independent vaccine strategies are urgently needed. To that end, several high-valency vaccines are now in development to meet this need. GSK inherited a 24-valent conjugate candidate (PCV24) through its acquisition of Affinivax but has recently halted its adult PCV24 program, citing “increased competition” and choosing to focus on a yet higher-valency version. Nonetheless, GSK continues a 24-valent program in children. Vaxcyte’s 31-valent conjugate (PCV31) has shown very encouraging early results with phase 1/2 data reporting the elicitation of functional antibodies against all 31 serotypes. Pfizer is also pursuing higher-valency PCVs: Public disclosures note a 25-valent candidate in phase 2 and a >30-valent program in preclinical development. These next-generation candidates aim to capture virtually all known serotypes that cause disease today (*16*).

As efforts intensify to expand serotype coverage through higher-valency conjugate vaccines, other innovative strategies have moved beyond polysaccharide targets together, instead exploring protein-based or conserved antigen approaches to address the challenges posed by pneumococcal diversity. It is well established that *S. pneumoniae* is a highly antigenically diverse pathogen, capable of adapting to different tissue environments and evading host immune responses. Over the past decade, substantial progress has been made in identifying pneumococcal virulence factors and understanding their variability across strains and disease states (*17*–*20*). Numerous vaccine candidates have been proposed using traditional strategies, such as targeting surface-exposed proteins or conserved oligosaccharides and selecting virulence factors implicated in disease severity. Among the most studied protein targets are pneumolysin (Ply) and its detoxified mutants (*21*), the choline-binding proteins PspA and CbpA (also known as PspC), PcpA, the metal-binding protein PsaA, the iron transporters PiuA/PiaA, and the histidine triad family proteins (*22*–*27*). Despite promising preclinical data, no protein-based pneumococcal vaccine has yet reached the market. For instance, the PhtD-pneumolysoid combination failed to demonstrate efficacy against carriage when tested alongside PCV13 (*28*) and was discontinued because of the prohibitive cost of large-scale efficacy trials. Other candidates, such as PspA, were withdrawn during clinical development because of safety concerns, including the risk of triggering autoimmune responses (*29*). Likewise, a whole-cell vaccine approach was abandoned after phase 1 studies reported unacceptable reactogenicity in children (NCT02543892). More recently, GPN Vaccines developed Gamma-PN, a nonadjuvanted whole-cell vaccine that has shown safety and immunogenicity in adults aged 50 to 69 years, with ongoing phase 1 evaluation in older adults (*30*).

In the present study, we applied an expanded reverse vaccinology framework to identify conserved pneumococcal antigens with potential for broad coverage. Reverse vaccinology was originally established through genome-led antigen discovery studies by Rappuoli *et al.* (*31*, *32*) and was subsequently adapted to pneumococcus by Wizemann *et al.* (*33*) and others (*34*, *35*). Building on these foundations, our approach integrates pangenome-scale comparative genomics (>20,000 pneumococcal genomes), multiparameter antigen prioritization, and downstream functional validation in translational murine models. We therefore view the present study as a scaled and experimentally validated advancement of established genome-guided antigen discovery approaches.

Our study led to the identification of three antigens: zinc metalloprotease B (ZmpB, SPD_0577), pneumococcal adherence virulence factor A (PavA, SPD_0854), and a YfhO-like transmembrane protein (SPD_2058). Together referred to as ZPY, these antigens were recombinantly expressed and formulated with a CpG oligonucleotide (a Toll-like receptor 9 ligand) and chitosan salt (a mucoadhesive adjuvant). The resulting formulation, i.e., ZPY-CpG-chitosan (ZPY-CpG-Ch), was evaluated in murine in vivo models. Notably, it demonstrated superior protection compared to PCV13, particularly against nonvaccine serotypes. Immunological analyses indicated a robust antigen-specific response, with elevated cytokine production and IgG subclass titers. A strong T helper 7 (T_H_17)–mediated immune response was observed, suggesting a key mechanism underlying the protective efficacy of this formulation. T_H_17-mediated immunity is well established as a critical component of host defense against *S. pneumoniae*, particularly in the context of protein-based vaccines (*29*, *36*–*39*). Together, our findings underscore the potential of conserved protein antigens, combined with rational adjuvant design, to deliver broad and durable protection against pneumococcal disease, offering a promising alternative to polysaccharide-based strategies.

## RESULTS

### Global distribution, serotype diversity, and population structure of pneumococcal isolates

In the initial steps, isolate genome assemblies were gathered from sequence databases and subjected to quality control, as described in Materials and Methods. Subsequently, 20,958 passing assemblies were used to create core single-nucleotide polymorphism (SNP) trees and to extract the protein sequences of the proteins of interest from each strain. To gain insights into the global distribution and genetic diversity of the pneumococcal strains in our dataset, we analyzed serotype distribution across different regions (fig. S1A), spanning six continents (Asia, Africa, North America, South America, Europe, and Australia) and 64 different countries. Geographic mapping revealed clusters of closely related strains within specific regions, underscoring the potential epidemiological impact of such groupings on vaccine coverage strategies.

Our analysis of core SNPs across strains yielded an average nucleotide identity of 98%, confirming a high degree of genetic relatedness across the dataset despite regional variation (fig. S2A). A minimum spanning tree further illustrated genetic relationships by country, highlighting geographical clustering and some degree of evolutionary separation (fig. S2B). This clustering supports the hypothesis that pneumococcal populations are highly structured yet geographically widespread (*40*). Population structure analyses using FastBAPS (levels 1 and 2) (*41*) and PopPUNK (*42*) were performed to provide complementary views of genetic diversity within the dataset (fig. S3). While these two clustering approaches showed only partial concordance, reflecting differences in underlying methodology, both analyses indicate that the dataset captures broad pneumococcal diversity.

A minimum spanning tree analysis of the 20,958 pneumococcal genomes highlighted extensive phylogenetic diversity across isolates (fig. S4), which reflects both the evolutionary pressures and diversity of *S. pneumoniae*. In addition, we analyzed the prevalence of pneumococcal genomes by serotype, categorized as vaccine or non-vaccine types (fig. S5). Our complete dataset of isolates comprised 96 distinct pneumococcal serotypes, with 1719 strains described as nontypable, variant, or inconclusive. This categorization offers insight into the dataset’s distribution across vaccine formulations, which was instrumental for ensuring our investigational vaccine’s anticipated coverage, particularly in addressing serotype replacement. We additionally noted particular clade branches within the dataset phylogeny, which were associated with multiple serotypes, countries, age groups, and infection sites (figs. S6 to S8), which attest to the biological relevance and breadth of our collection.

### Vaccinology pipeline and candidate antigen selection

**Fig. 1.**
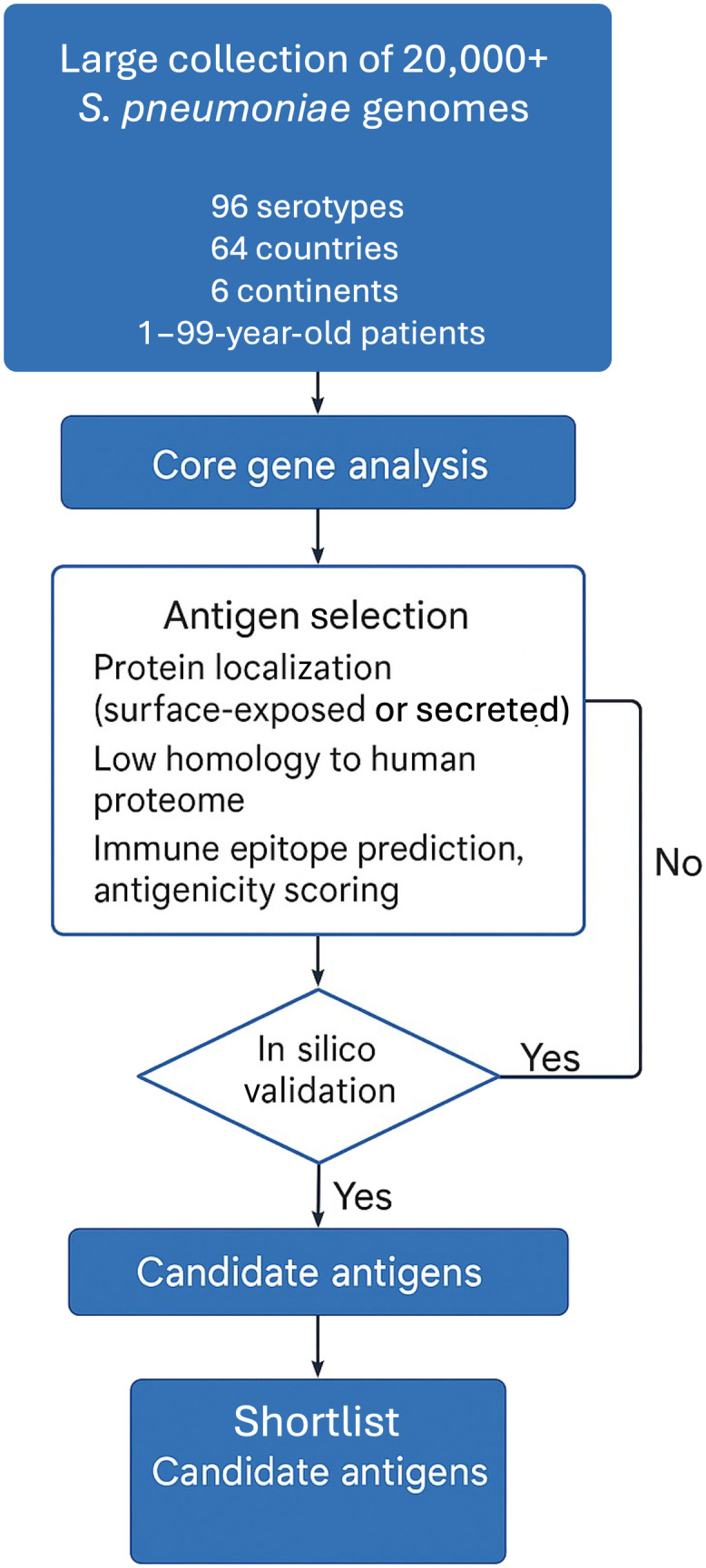
Analytical pipeline used for the rational discovery of universal pneumococcal antigens via pangenome reverse vaccinology. This figure illustrates the analytical pipeline used for identifying universal pneumococcal antigens by using genome-wide reverse vaccinology. The process begins with accession to publicly available large-scale collection of *S. pneumoniae* genomes (*n* = 20,958), followed by bioinformatic analysis to identify conserved genes across multiple strains. Core gene analysis is conducted to select antigens with minimal variability and broad coverage. Potential vaccine targets are further filtered on the basis of protein localization (surface exposed or secreted), low homology to the human proteome, as well as immune epitope prediction, and antigenicity scoring. Last, candidate antigens undergo in silico validation for their instability index (ProtParam) and predicted three-dimensional conformational (AlphaFold, DeepMind) properties.

We sought to develop a comprehensive analytical pipeline for the discovery of universal pneumococcal antigens using pangenome reverse vaccinology to address the high genetic variability among *S. pneumoniae* strains (workflow depicted in Fig. 1). Our approach began with collating an unprecedentedly large number (20,958) of publicly available genome sequences, followed by bioinformatic filtering and quality control to retain 15,986 high-quality pneumococcal assemblies. To enable computational scalability across this dataset, genomes were clustered into representative groups, and pangenome analysis of 34 cluster representatives was performed using Panaroo. Core genome analysis identified 1301 conserved genes across the dataset, consistent with prior pneumococcal pangenome studies (*43*–*45*). Given the open-ended nature of the pneumococcal pangenome—wherein the core gene set can only decrease as further genomes are incorporated (*46*)—this figure represents a conservative and robust estimate. Filtering based on predicted subcellular localization (PSORTb 3.0), signal peptide type (SignalP 5.0), membrane topology (DeepTMHMM), and adhesin probability (SPAAN) reduced this to 21 candidate proteins enriched for cell wall–localized or surface-exposed antigens. Subsequent prioritization based on antigenicity (Vaxign) and low homology to the human proteome (BLASTp), using a composite scoring rubric (maximum score of 6), resulted in 15 high-confidence candidates achieving scores of ≥5. Three lead antigens were then selected on the basis of published evidence for roles in pneumococcal virulence and pathogenesis, as well as prior experimental characterization within our group, providing an additional layer of biological validation supporting their advancement to recombinant protein production and in vivo evaluation. This systematic method aligns well with established reverse vaccinology frameworks successfully used with bacterial pathogens such as *Neisseria meningitidis* (*32*, *47*) and also tested with others (*48*, *49*), but distinguishing features here are the scale of the genomic dataset analyzed, the integration of multiple prioritization filters within a single pipeline, and the direct progression from in silico candidate selection to recombinant protein production and functional in vivo evaluation.

Using our workflow, we identified a set of three genes of interest, namely, zinc metalloprotease B (*zmpB*; National Center for Biotechnology Information gene ID: 7680834), pneumococcal adherence virulence factor A (*pavA*; gene ID: 12889101), and YfhO-like transmembrane protein (*yfhO-like*; gene ID: 13695552). ZmpB protein contains the N- and C-terminal domains of peptidase M26, a peptidase linked to the ability to cleave human IgA and often attached to the cell wall (*50*). ZmpB was reported to be essential for pneumococcal virulence, with knockout strains showing reduced bacterial load and improved survival (*51*). The absence of *zmpB* in *S. pneumoniae* infections results in significantly reduced levels of tumor necrosis factor–α in the lungs, suggesting its role in inflammatory events leading to bacterial translocation (*52*). PavA is a surface-exposed fibronectin-binding protein in *S. pneumoniae* that plays a critical role in host colonization and disease progression (*53*). Functional studies have shown that *pavA*-deficient mutants exhibit significantly reduced adherence and invasion capabilities in vitro and in murine models (*54*, *55*). Our third gene candidate is a transmembrane protein that is a member of the YfhO superfamily, transmembrane proteins essential to lipoteichoic acid glycosylation. In InterPro, the protein family is described as having an unknown function, transcribed in sporulation medium, and regulated by a two-component system, YvrGHb, in *Bacillus*. InterPro additionally mentions that some members of the family have been annotated as putative ATP (adenosine 5′-triphosphate)–binding cassette (ABC) transporter permease proteins. However, within our transmembrane protein of interest, any ABC transport domain is lacking. While these three protein candidates have been previously described individually, they have not, to our knowledge, been evaluated in combination within a single vaccine formulation. The novelty of this study lies in the rational combination of these conserved antigens, supported by large-scale genomic prioritization across >20,000 pneumococcal genomes and subsequent functional validation in vivo.

### Genomic conservation and structural characterization of candidate antigens

**Fig. 2.**
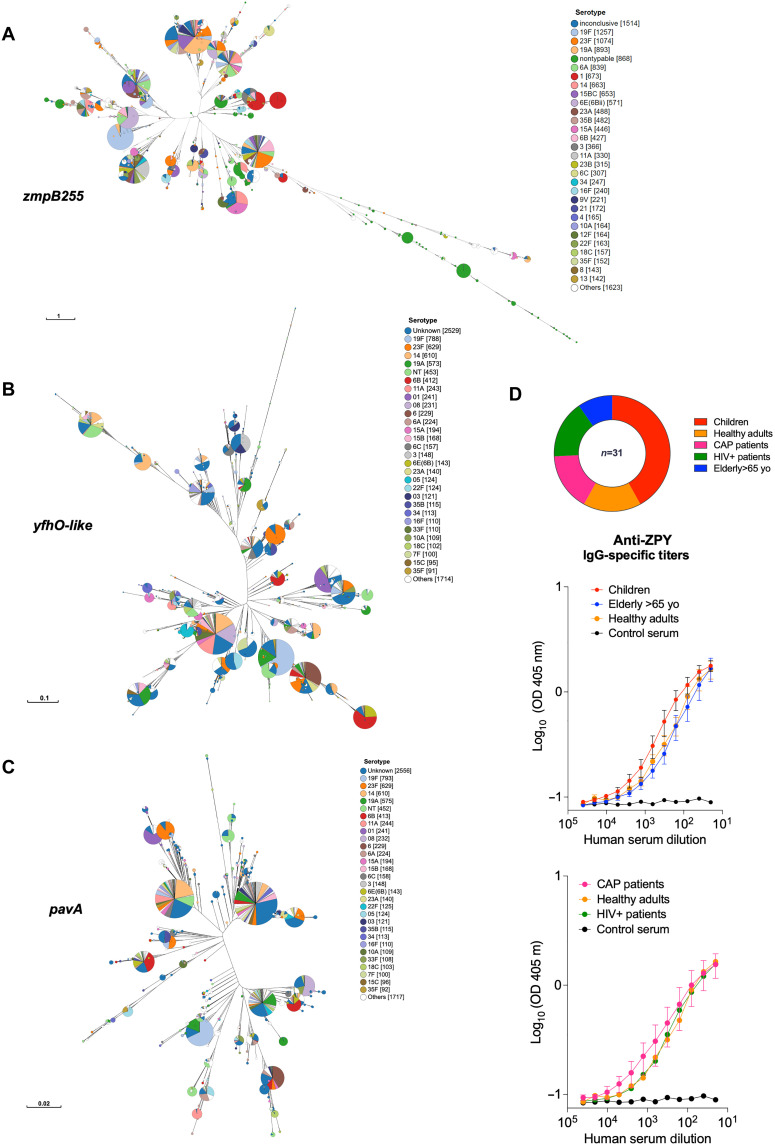
Genetic variability of *zmpB*, *pavA*, and *yfhO*-*like* across pneumococcal serotypes and their potential as universal vaccine targets. The combination of all three proteins is coined ZPY. (**A** to **C**) Minimum spanning trees visualizing the genetic relationships among serotypes on the basis of variations in the *zmpB* (A), *yfhO-like* (B), and *pavA* (C) genes, the three candidates selected for vaccine development in this study. Each node represents a genome cluster, and the branches show the genetic distances between them. Different serotypes are distinguished by color, highlighting how *ZmpB*, *YfhO-like*, and *PavA* allelic variants are distributed across serotypes. Clusters indicate closely related serotypes with similar gene sequences, while more distantly related clusters are separated by longer branches, reflecting greater genetic divergence. (**D**) Serum IgG antibody response to the three pneumococcal candidate antigens in healthy and at-risk patient groups. This figure illustrates the IgG antibody binding activity against the three pneumococcal antigens of interest in sera from different subject groups. Antigens were coated onto high-binding ELISA plates to assess the specific IgG responses. The groups included children aged 24 months to 7 years (red circles), healthy adults (orange circles), adults with CAP (pink circles), HIV-positive adults with no history of pneumococcal infection (green circles), and elderly patients (dark blue circles). The data represent the mean OD at 450 nm +/− SEM values for each group. A total of 31 serum samples were analyzed: *n* = 13 children, *n* = 5 healthy adults, *n* = 5 adults with CAP, *n* = 5 HIV-positive adults with no history of pneumococcal infection, and *n* = 3 elderly patients.

Next, we examined the species-wide distribution of our three in silico–validated antigen candidates. Gene presence-absence profiling (fig. S1B) revealed that *pavA* and the *YfhO-like* gene were universally conserved across all isolates, regardless of lineage or geographical origin—strongly supporting their potential as broadly protective vaccine targets given their essential roles in adherence and cell-surface architecture. In contrast, *zmpB* showed a variable distribution, consistent with reports of strain-dependent virulence effects. However, a notable finding emerged: The N-terminal 255-amino-acid segment of ZmpB was present in all pneumococcal genomes included in our analysis. This pattern suggests that while *zmpB* as a whole is variable, its conserved N-terminal region (*zmpB255*) may represent a stable antigenic motif, offering a useful benchmark for considering whether incorporating antigenic diversity could broaden immune coverage against *S. pneumoniae*. Minimum spanning tree visualizations (Fig. 2, A to C) were used to represent genetic relationships and population structure among isolates, providing an overview of the distribution of antigen variants across the dataset. Quantitative evidence for antigen conservation is presented in Table 1, including sequence identity and prevalence metrics. Together, these analyses indicate that the selected antigens are conserved across a genetically diverse pneumococcal population, supporting their potential suitability for serotype-independent vaccine design.

**Table 1.** *S. pneumoniae* vaccine candidates: ZmpB, PavA, and Yfho-*like* (ZPY) antigens.

Locus_tag*	Gene name	Product	Conservation^†^	Instability index
SPV_0854	*pavA*	Fibronectin/fibrinogen binding protein	99.0 ± 0.27%	47.62468
SPV_0577	*zmpB*	Zinc metalloprotease ZmpB precursor, N-terminal 255 amino acids	96.7 ± 1.15%	36.94745^‡^
SPV_2058	*yfhO-like*	Transmembrane protein member of YfhO superfamily	99.4 ± 0.35%	32.43708

* Reference strain *S. pneumoniae* D39V, complete genome.

† Calculated as the mean percentage of amino acid identity ± the standard deviation across a collection of 113 complete finished *S. pneumoniae* genome assemblies.

‡ ZmpB complete sequence instability index: 36.18018.

Additional analyses further assessed antigen suitability across various epidemiological contexts. The candidate genes were consistently detected across isolates spanning samples collected between 1941 and 2024 (fig. S6), indicating stable representation within the pneumococcal population. While this analysis does not assess temporal dynamics, it confirms that the selected antigens are broadly maintained across isolates sampled over different time periods, consistent with previous observations that core genomic elements in *S. pneumoniae* remain relatively stable over time (*40*, *56*). These results suggest that a vaccine formulation composed of ZmpB, PavA, and YfhO*-like* encoding genes (ZPY) or their products could provide durable protection even amid shifts in serotype prevalence. Age-specific analyses of genetic diversity revealed distinct patterns of strain prevalence across different age groups, underscoring the need for age-appropriate vaccine coverage to maximize efficacy across populations (fig. S7). In addition, site-specific clustering of strains by anatomical isolation sites may inform the design of ZPY-based vaccine formulation to enhance protection across various infection sites (fig. S8). In-depth amino acid alignments of PavA, YfhO-*lik*e, and ZmpB identified both conserved and variable regions (figs S9 to S11).

To further characterize the structural diversity of ZmpB, we interrogated the amino acid alignment of its N-terminal 255 residues across a large representative dataset of 15,979 pneumococcal genomes. This region was notably conserved in all isolates, with divergence emerging only beyond this point in the form of variable repeat domains within the central portion of the protein. The C-terminal region also showed moderate conservation but separated into two clearly distinguishable sequence types. Within the alignment, two major ZmpB255 variants—MAPS and FAPS—were evident (fig. S12). All isolates carried the MAPS variant, while only two strains additionally encoded the FAPS form. Notably, strains AMS235799v1 and 55312 contained only the MAPS variant, with near-identical sequence identity up to residue 86. Together, these data demonstrate that ZmpB combines a highly conserved N-terminal domain with structured variation in downstream regions, a pattern that may influence its functional role in pneumococcal virulence and its suitability as an antigenic component in future vaccine designs.

### Measurement of IgG antibody titers against ZPY antigens in healthy adults and disease-susceptible groups

A total of 31 serum samples were collected from different demographic and clinical groups, including young children (aged 24 months to 9 years), healthy adults, adults with community-acquired pneumonia (CAP), HIV-positive adults without prior pneumococcal infection, and elderly patients. These samples were assayed to detect and quantify specific IgG antibodies against the protein antigens ZmpB, PavA, and YfhO-*like* (Fig. 2D). When the assay plate was coated with all three antigens simultaneously (Fig. 2D), serum IgG responses to the pneumococcal antigens varied across the different groups. Enzyme-linked immunosorbent assay (ELISA) quantified IgG binding, revealing distinct responses. Optical density (OD) at 450 nm and area-under-the-curve (AUC) measurements indicated that antibody levels were higher in children (4511 ± 311.9; *n* = 13) compared to healthy adults (4153 ± 202.0; *n* = 5) and elderly (3896 ± 88.39; *n* = 3) groups, although this difference was not statistically significant, suggesting potential age-related variations in pneumococcal immunity. Adults with CAP (4619 ± 370.4; *n* = 5) showed a higher IgG level trend compared to HIV-positive adults (4022 ± 95.33; *n* = 5), suggesting a more robust immune activity during active infection, consistent with earlier reports (*57*, *58*). Our findings highlight that both age and underlying health status influence baseline immunity to pneumococcal antigens, emphasizing potential immunity gaps among young children and elderly populations. When antibody levels against the three proteins were measured separately (fig. S13), AUC titers were consistently and statistically significant higher against ZmpB across all patient groups compared to YfhO-*like* and PavA. This may be attributed to its larger molecular size and globular structure. The anti-PavA antibody AUC titers were more elevated in the elderly (3587 ± 86.42; *n* = 3) and HIV-positive adult (3561 ± 149.1; *n* = 5) groups compared to other groups, suggesting a possible role for PavA in the pathogenesis of pneumococcal disease in these patient groups. PavA’s function in adhesion and host interaction may contribute to immune recognition, making it a suitable target for vaccination, particularly for immunocompromised individuals who are more susceptible to severe pneumococcal disease (*59*). The anti–Yfho-*like* antibody titers did not vary significantly between patient groups. In healthy adults, the natural antibody response followed the pattern ZmpB > YfhO-like > PavA (4550 ± 151.5 versus 3586 ± 78.15 versus 3487 ± 54.18, respectively, *n* = 5).

### Immunization with ZPY-CpG-Ch confers dose-responsive protection against *S. pneumoniae* invasive disease

**Fig. 3.**
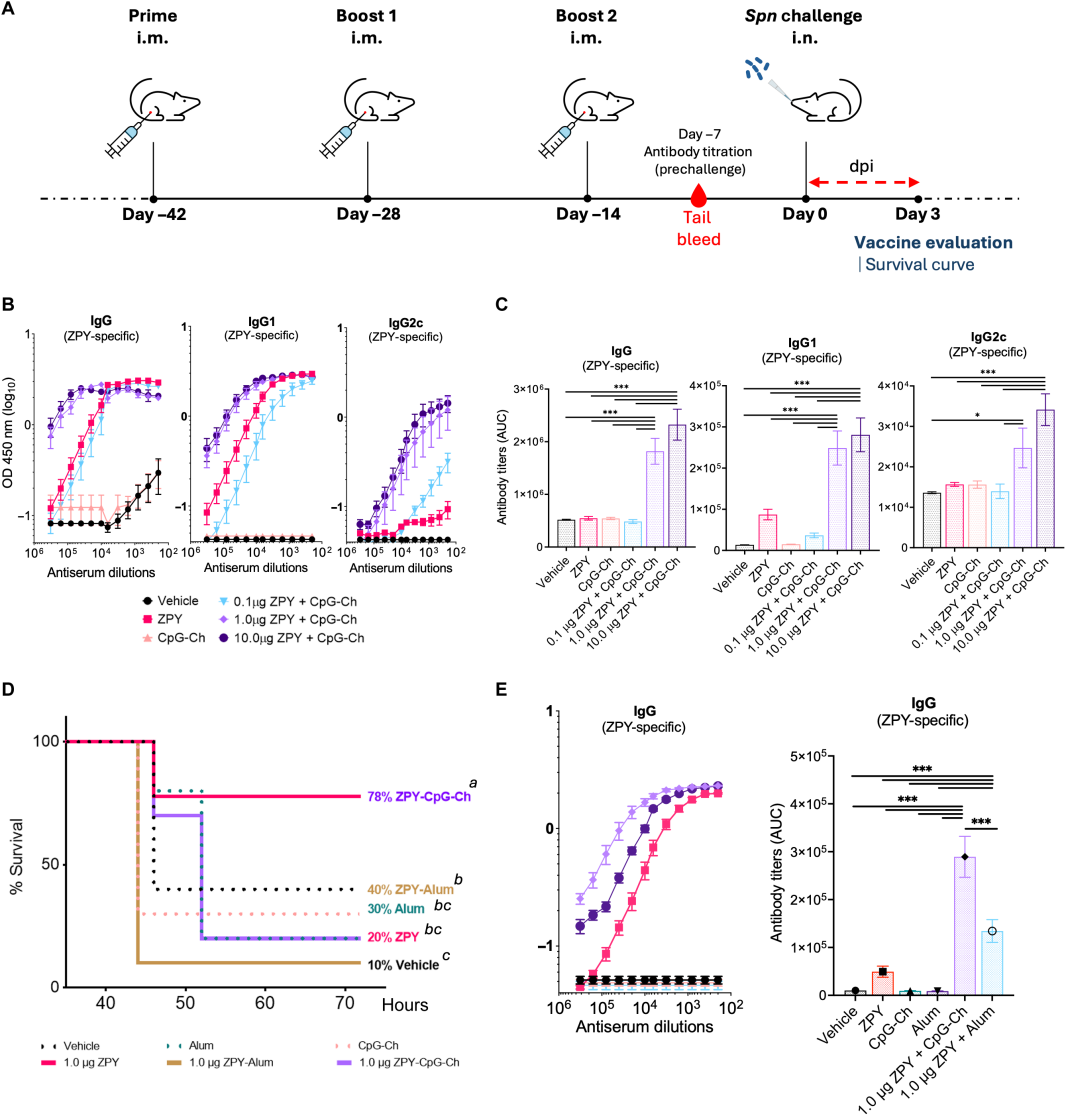
Immunization with ZPY-CpG-Ch confers dose-responsive protection against *S. pneumoniae* invasive disease. (**A**) Schematic representation of the immunization schedule for 6- to 8-week-old C57BL/6J mice. Mice received three doses at 2-week intervals, followed by a challenge 14 days after the final dose. i.m., intramuscularly; i.n., intranasally; dpi, days postinfection. (**B** and **C**) Antigen-specific IgG, IgG1, and IgG2c antibody titers were measured in mice administered varying doses of ZPY, ranging from 0.1 to 10 μg per dose. Antibody levels are shown as OD at 450 nm (B) and AUC (C). (**D** and **E**) Vaccination with the ZPY-CpG-Ch formulation offered superior protection against invasive disease compared to the Alum-adjuvanted formulation. Mice were vaccinated with PBS (vehicle), adjuvant alone (Alum or CpG-Ch), or adjuvant and antigen (1.0 μg of ZPY + Alum or 1.0 μg of ZPY + CpG-Ch). Two weeks after the final dose, mice were intranasally challenged with 5 × 10^5^ CFU of a hypervirulent pneumococcal serotype 1 strain and monitored for disease symptoms. (D) Survival was recorded using Kaplan-Meier analysis. (E) IgG antibody titers postvaccination were measured as OD units (left panel) and AUC (right panel). Data are presented as the means ± SEM, *n* = 10 per group. Statistical significance was assessed using the log-rank (Mantel-Cox) test for survival, with different letters indicating significant differences. The Kruskal-Wallis test with Dunn’s multiple-comparisons post test was used for IgG titers. Significance levels: *P < 0.05 and ***P < 0.001.

A CpG-Ch vaccine formulation was selected on the basis of its combined immunostimulatory and delivery properties. CpG induces T_H_1- and T_H_17-associated responses, while chitosan enhances antigen uptake and cellular immunity. Immunization with ZPY combined with CpG-Ch demonstrated dose-dependent protection against *S. pneumoniae* in C57BL/6J mice. Following an immunization regimen consisting of three doses administered bimonthly, mice were challenged intranasally with 5 × 10^5^ colony-forming units (CFU) of a hypervirulent serotype 1 strain, 2 weeks after the final dose (Fig. 3A).

Serum analyses revealed that ZPY in combination with CpG-Ch elicited robust antigen-specific IgG1 and IgG2c responses, which were both dose-dependent (Fig. 3, B and C). The OD values at 450 nm and AUC measurements indicated significantly different antibody titers in a dose-dependent manner with AUC titers 0.1 μg >1.0 μg >10 μg in mice vaccinated with ZPY formulated with CpG-Ch (0.1 μg at 36,484 ± 5686 compared to 10 μg at 280,733 ± 41,002). This dose-response effect aligns with findings by other studies, which reported enhanced immunogenicity and greater protective efficacy when using CpG as an adjuvant to stimulate both humoral and cellular immunity (*60*). The survival rate was also markedly higher in groups immunized with the ZPY-CpG-Ch formulation compared to those given ZPY formulated with Alum or adjuvant alone (adjuvant-alone AUC at 9375 ± 464.8 versus 1.0 μg of ZPY-Alum AUC titers at 134,318 ± 23,818 versus 1.0 μg of ZPY-CpG-Ch AUC at 289,312 ± 42,836). Kaplan-Meier survival analysis (Fig. 3D) illustrated that mice vaccinated with ZPY-CpG-Ch experienced significantly increased survival rates (78%) postchallenge compared to vehicle (10%), ZPY-only (20%), and ZPY-Alum (40%) groups (log-rank Mantel-Cox test, *P* < 0.001). Partial protection (30%) observed in the Alum adjuvant–only group likely reflects nonspecific innate immune activation rather than antigen-specific adaptive immunity. This is consistent with the well-established immunostimulatory properties of Alum (*61*).

Upon measuring ZPY-specific IgG antibody titers (Fig. 3E) in sera collected from mice at the experimental end point, we observed that mice immunized with ZPY-CpG-Ch developed the highest antibody concentrations (289,312 AUC ± 42,836), which were ∼5.8 times higher than those immunized with ZPY antigen only (49,465 AUC ± 11.569) and 2.2 times as high as the Alum-adjuvanted group (8871 AUC ± 314.7). These differences were highly statistically significant.

Our findings support CpG-Ch as a potent adjuvant choice that can augment the protective effects of protein-based pneumococcal vaccines. Previous studies have underscored the limitations of traditional Alum adjuvants, as they primarily stimulate humoral immunity without strongly activating cellular responses, which are crucial for combating microbial pathogens and achieving comprehensive immunity (*62*). The use of CpG oligodeoxynucleotide as a Toll-like receptor 9 agonist in this study likely contributed to a broader immune activation profile, providing stronger and more sustained protection than Alum alone. These results position ZPY formulated with CpG-Ch as a promising candidate for broader pneumococcal protection, offering an alternative to traditional conjugate vaccines that may address their inherent serotype limitations. We carried out additional analysis comparing the full ZPY-CpG-Ch formulation to single-antigen formulations: Our results revealed superior protective efficacy, with significantly improved survival outcomes, reduced bacterial burden in the lungs and blood (fig. S14, A and B), and enhanced antibody responses (fig. S14, C and D) in mice receiving the tripartite ZPY vaccine formulation. A prominent cellular immune response characterized by the production of interferon-γ (IFN-γ) and interleukin-17A (IL-17A) was evidenced in mediastinal lymph node (MLN) ex vivo cultures (fig. S14E). The stronger immune responses observed with the trivalent formulation are likely multifactorial. While the total antigen dose is higher in the combined formulation, the presence of multiple antigens may also increase epitope diversity, enhance antigen presentation, and promote synergistic activation of T cell responses. Similar effects have been reported in multicomponent pneumococcal protein vaccines, where combined antigens induce stronger T_H_1/T_H_17 responses than individual components (*63*, *64*). Given the established role of IL-17A–producing CD4^+^ T cells in pneumococcal immunity (*29*, *36*), these findings provide a plausible mechanistic basis for the enhanced protection observed.

### Protective efficacy of ZPY formulated with CpG-Ch is noninferior to PCV13 in murine models of pneumococcal pneumonia

The protective efficacy of ZPY formulated with CpG-Ch (ZPY-CpG-Ch) was compared to PCV13 in murine models of pneumococcal pneumonia, evaluating both adult and neonatal mice. Adult mice were immunized with either PCV13, Alum alone, CpG-Ch alone, ZPY alone, ZPY combined with CpG-C, or ZPY combined with Alum. Each ZPY-based formulation contained 1.0 μg of each of the three target antigens. At 14 days following the second booster immunization, mice were challenged intranasally with 5 × 10^5^ CFU of a hypervirulent *S. pneumoniae* serotype 1 strain.

**Fig. 4.**
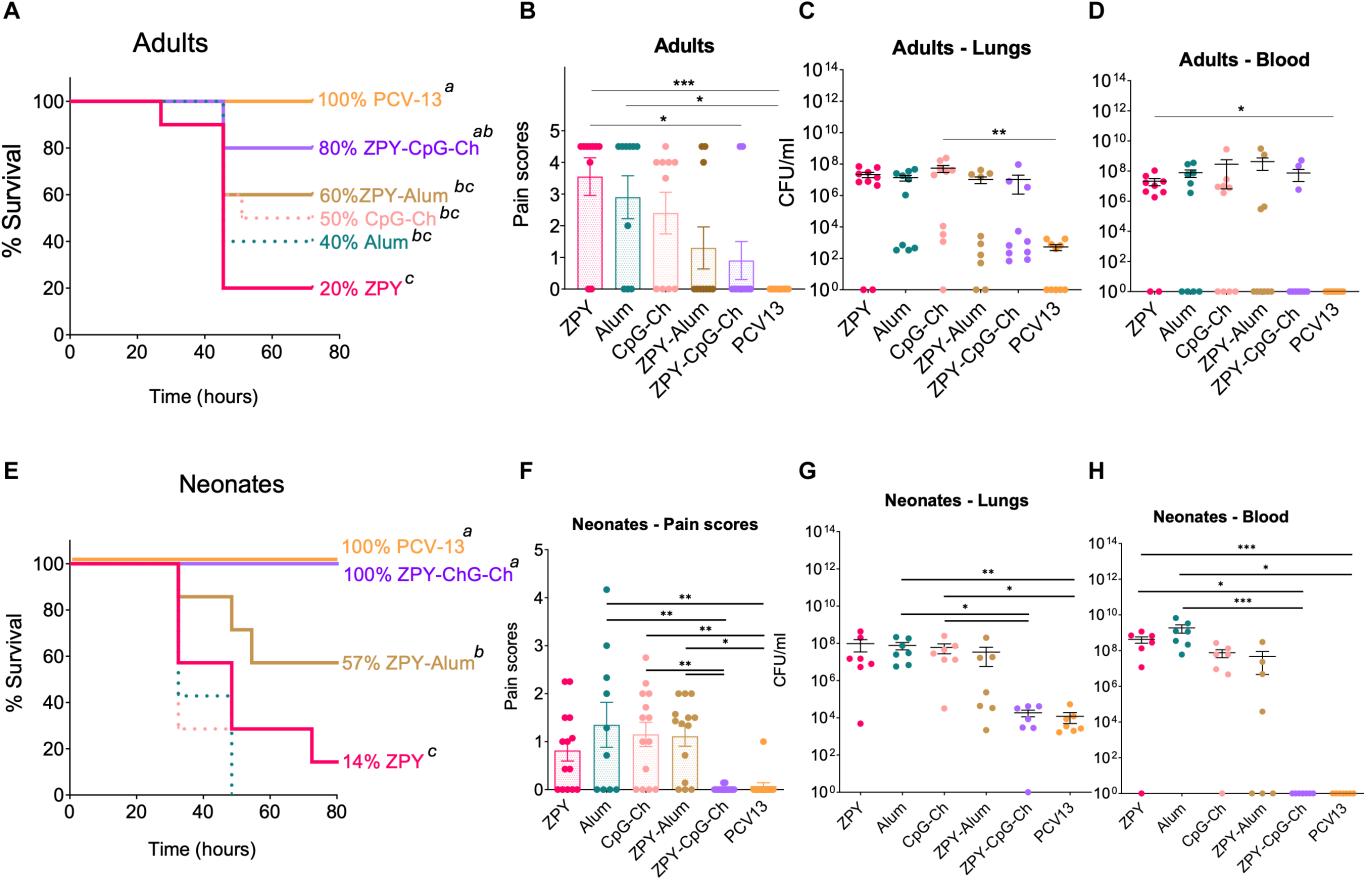
Protective efficacy of ZPY-CpG-Ch compared to PCV13 in murine models of pneumococcal pneumonia in adults and neonate mice. This figure evaluates the efficacy of ZPY-CpG-Ch in comparison to PCV13 in adult and neonate mouse models. Adult mice (*n* = 10 per group) were immunized with either PBS (vehicle), Alum, CpG-Ch, ZPY alone, ZPY-CpG-Ch, or ZPY-Alum, where each formulation contained 1.0 μg of each of the three antigens per dose. (**A** and **B**) Two weeks after the final dose, mice were intranasally challenged with 5 × 10^5^ CFU of a hypervirulent pneumococcal serotype 1 strain. Survival rates were recorded via Kaplan-Meier curves (A), and clinical symptoms were monitored (B). (**C** and **D**) Bacterial load was quantified by determining CFU in lung (C) and blood (D) samples. For the neonate study, mice were immunized starting at 7 days old (prime), followed by two weekly boosters. (**E** to **H**) Survival (E), clinical symptoms (F), lung CFU counts (G), and blood CFU counts (H) were similarly recorded in these young animals. Data are presented as the means ± SEM, with *n* = 10 for adult mice and *n* = 7 for neonates. Statistical analysis: Log-rank (Mantel-Cox) test for survival and Kruskal-Wallis test with Dunn’s post test for CFU counts. Significance levels: *P < 0.05; **P < 0.01; ***P < 0.001. Different letters indicate significant differences.

In adult mice, survival analysis (Fig. 4A) revealed that ZPY formulated with CpG-Ch led to a significantly higher survival rate (80%) compared to control and other adjuvant groups, with Kaplan-Meier curves showing improved survival over the ZPY-alone (20%) and ZPY-Alum (60%) groups (log-rank Mantel-Cox test, *P* < 0.001). The CpG-Ch–only group showed intermediate protection compared with vehicle, suggesting that the adjuvant formulation itself may transiently enhance resistance to infection. This is consistent with the known immunostimulatory properties of CpG (*65*, *66*) and chitosan (*67*), which can enhance early host defense mechanisms (*68*). However, the superior survival and reduced bacterial burden observed in the ZPY-CpG-Ch group demonstrate that antigen inclusion is required for optimal protection.

In line with these observations, the clinical symptoms, recorded alongside survival, were notably less severe in the ZPY-CpG-Ch group (Fig. 4B, ZPY-CpG-Ch, *P* < 0.05). This enhanced protection was further evidenced in the bacterial densities, with CFU in blood samples (Fig. 4, C and D) being detected in fewer animals or at a lower density in the ZPY-CpG-Ch group. While the absolute survival rates differed (80% for ZPY-CpG-Ch versus 100% PCV13), no statistically significant difference was observed between the groups (log-rank Mantel-Cox test, *n* = 5 to 10 mice per group), supporting the conclusion that ZPY-CpG-Ch is noninferior to PCV13 under these experimental conditions.

In neonates, survival outcomes mirrored those in adults. Starting at 7 days of age, neonatal mice received primary immunization followed by two weekly boosters. Postchallenge, neonates immunized with ZPY-CpG-Ch displayed significantly higher survival rates (Fig. 4E) and significantly reduced clinical symptom severity (Fig. 4F) compared to ZPY-alone (14%), ZPY-Alum (57%), and other adjuvant–only groups (log-rank Mantel-Cox test, *P* < 0.001). Lung and blood CFU counts (Fig. 4, G and H) were also substantially lower in the ZPY-CpG-Ch group, showing increased protection compared to the adult models and underscoring the robust immunogenicity of this formulation across age groups. Most notably, neonate immunization with ZPY-CpG-Ch was noninferior to PCV13, as both groups showed 100% survival rates and nearly identical CFU counts in the lungs and blood.

To further understand the immunological kinetics underlying the observed protection, we tracked antigen-specific IgG responses across the prime-boost-boost regimen in both neonatal and adult mice. ELISA analyses revealed progressive increases in antibody titers following each immunization, with markedly higher responses in the ZPY-CpG-Ch group compared to ZPY alone or ZPY-Alum (fig. S15).

### ZPY-CpG-Ch formulation enhances protective efficacy against non-PCV13 serotypes

The protective efficacy of ZPY formulated with CpG-Ch was tested against non-PCV13 pneumococcal serotypes 8, 11A, and 33F—these serotypes were documented as prevailing non-PCV13 serotypes associated with severe disease, especially in high-income countries (*8*, *69*)—in a murine pneumococcal pneumonia model. Mice aged 6 to 8 weeks were immunized with ZPY (1.0 μg per antigen) formulated with CpG-Ch or with PCV13 as a benchmark and then challenged intranasally with 5 × 10^5^ CFU per mouse of each non-PCV13 serotype. Survival outcomes were tracked over a 3-day period, alongside pain scores to gauge disease severity and bacterial loads in lung and blood samples to assess bacterial clearance.

**Fig. 5.**
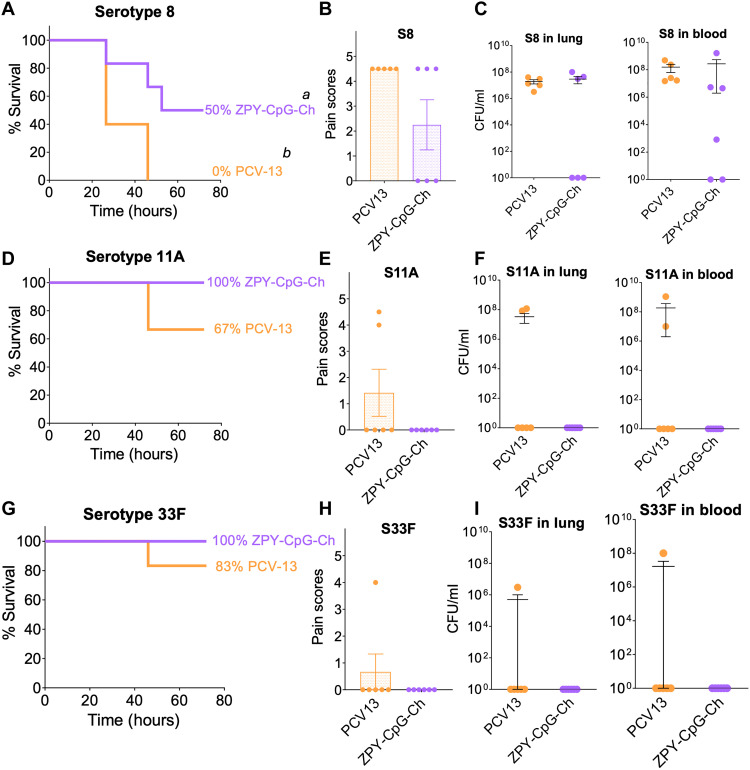
ZPY-CpG-Ch formulation enhances protective efficacy against non-PCV13 serotypes. Six- to 8-week-old mice were immunized with ZPY (1.0 μg per antigen) formulated with CpG-Ch or PCV13 and challenged with non-PCV13 pneumococcal serotypes (*S. pneumoniae* serotypes 8, 11A, and 33F) at a dose of 5 × 10^5^ CFU per mouse administered intranasally. Mice survival was monitored over a 7-day period following challenge. (**A**, **D**, and **G**) The percentage of surviving mice is plotted for each serotype (*n* = 5 or 6 per group). (**B**, **E**, and **H**) Pain scores were recorded postchallenge to assess disease severity. (**C**, **F**, and **I**) Viable pneumococcal counts were measured in lung and blood samples collected at the time of death. Data are shown as the means ± SEM, with *n* = 10 mice per group. Baseline control groups (vehicle and adjuvant only) are not included in this figure—see Fig. 3 for control groups data. This figure focuses on relative comparisons between vaccine groups, including benchmarking against PCV13. Statistical analyses were performed using the log-rank (Mantel-Cox) test for survival (*n* = 6 per group, except PCV13, *n* = 5); pain scores were analyzed using the Mann-Whitney nonparametric unpaired *t* test. Significance level: *P < 0.05; **P < 0.01; ***P < 0.001. Different letters indicate significant differences.

Mice vaccinated with ZPY-CpG-Ch demonstrated improved survival rates across all tested serotypes compared to the PCV13 group (Fig. 5, A, D, and G; serotype 8, *P = 0.0395; serotype 11A, *P* = 0.1380; serotype 33F, *P* = 0.3173), indicating broader protective efficacy than PCV13. Differences in observed protection across serotypes may reflect inherent variation in virulence and pathogenicity. In particular, serotype 8 is recognized as highly invasive (*70*–*72*), which may influence vaccine efficacy readouts in experimental models and contribute to the lower survival rates observed relative to other serotypes tested. Partial protection observed with PCV13 against nonvaccine serotypes is consistent with cross-reactive antibody responses and opsonophagocytic activity directed against structurally related pneumococcal epitopes, as reported previously (*73*). Pain scores (Fig. 5, B, E, and H) were lower in the ZPY-CpG-Ch group, correlating with reduced disease severity (*P* = 0.0756 in serotype 8, *P* = 0.1756 in serotype 11A, and *P* = 0.3632 in serotype 33F). Furthermore, bacterial counts in lung and blood samples collected at the time of death or upon study conclusion (Fig. 5, C, F, and I) showed a reducing trend in pneumococcal loads in ZPY-CpG-Ch–immunized mice, indicating enhanced bacterial clearance and lower systemic dissemination compared to PCV13. These findings underscore the potential of ZPY-CpG-Ch to provide broader serotype coverage beyond that achieved with conjugate vaccines limited to predefined capsular types.

### ZPY-CpG-Ch induces antibodies in mice that confer passive immunity and mediate opsonophagocytic activity

**Fig. 6.**
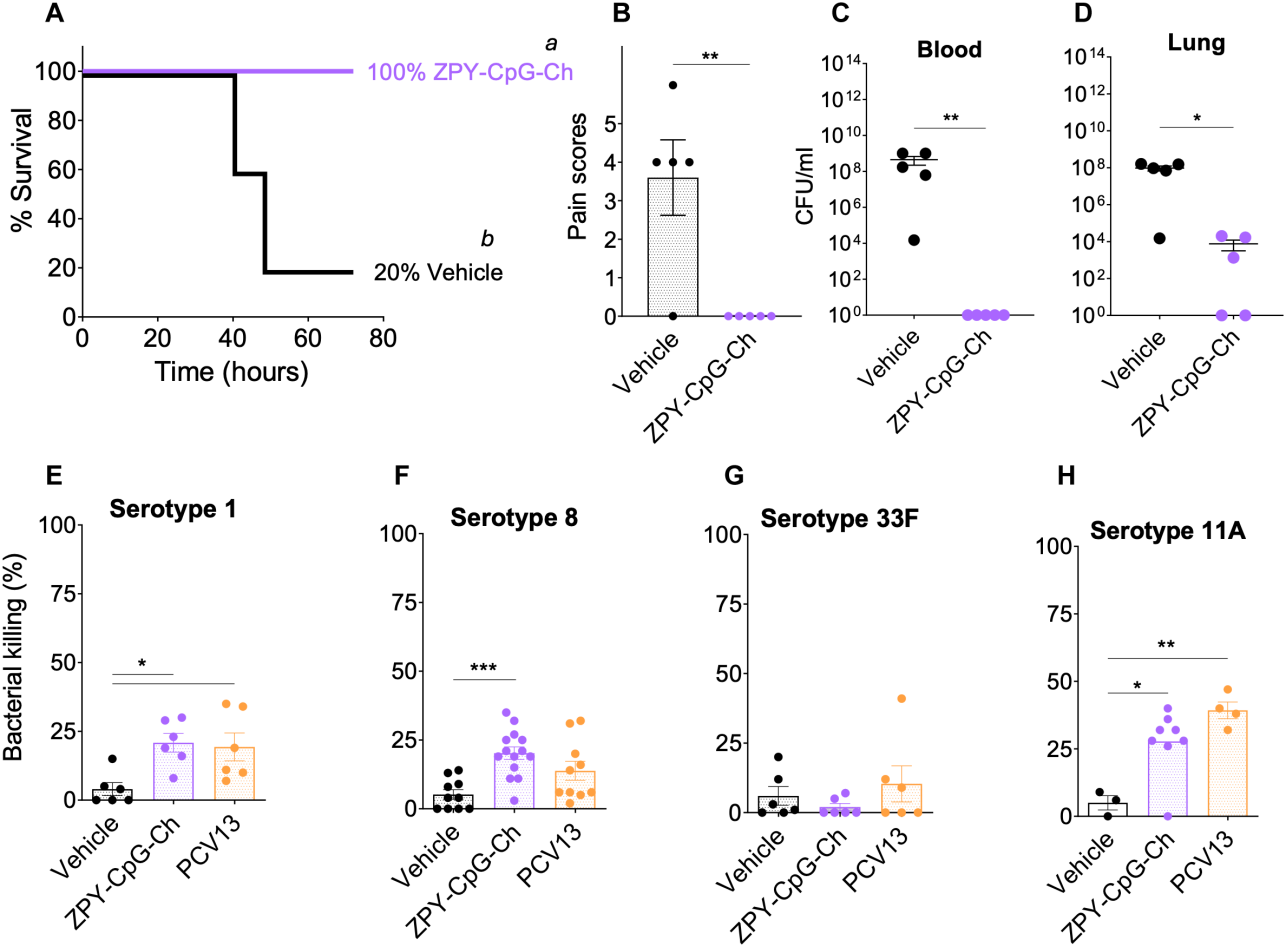
Passive immunity conferred by ZPY-CpG-Ch–induced mouse antibodies and their activity in in vitro OPK assays. Adult mice (*n* = 5 to 10 per group) were immunized with either a vehicle or a ZPY-CpG-Ch vaccine formulation containing YfhO-*like*, PavA, and ZmpB adjuvanted with CpG-Ch. Following immunization, the mice were culled via cardiac puncture, and their sera (100 to 150 μl per mouse) were transferred intravenously into naïve recipient mice. Four hours posttransfer, the passively immunized mice were intranasally challenged with hypervirulent serotype 1 *S. pneumoniae* in a pneumonia model. Survival times of the mice (**A**) as well as pain scores (**B**) and viable CFU counts in blood (**C**) and lung (**D**) samples were recorded. In addition, mouse sera (100 to 150 μl per mouse, diluted 1:16) were pooled for use in a functional HL-60–based OPK assay to evaluate the ability of the vaccinated mouse antiserum to opsonize viable pneumococci and mediate bacterial killing (**E** to **H**) of *S. pneumoniae* serotypes 1, 8, 11, and 33F. Statistical analyses were performed using the log-rank (Mantel-Cox) test for survival; CFU were compared using a nonparametric *t* test (Mann-Whitney test), and OPKs were compared using the Kruskal-Wallis test with Dunn’s test. Significance levels: *P < 0.05; **P < 0.01; ***P < 0.001. Different letters indicate significant differences.

Passive immunity was evaluated by transferring sera from immunized mice into naïve recipients, which were subsequently challenged with hypervirulent *S. pneumoniae* serotype 1. Following immunization, sera from adult mice vaccinated with either vehicle or ZPY-CpG-Ch were collected and transferred intravenously to naïve recipient mice, with 100 to 150 μl of serum per recipient. Four hours posttransfer, recipient mice were intranasally challenged in a pneumococcal pneumonia model, and survival outcomes were monitored alongside bacterial loads in blood and lung samples. Survival analysis indicated that mice passively immunized with sera from ZPY-CpG-Ch–immunized donors exhibited significantly prolonged survival times compared to controls (100% versus 20%, *P* = 0.0170; Fig. 6A). Furthermore, clinical scores (Fig. 6B) and viable CFU counts in blood (Fig. 6C) and lung (Fig. 6D) samples were significantly lower in this group, supporting the protective role of ZPY-CpG-Ch–induced antibodies in combating pneumococcal infection. To confirm the functional capacity of these antibodies, pooled sera from immunized mice were tested in an HL-60–based opsonophagocytic killing (OPK) assay. Sera (diluted 1:16) facilitated effective opsonization and bacterial killing of *S. pneumoniae*, including serotype 1 and nonvaccine serotypes such as 8 and 11A (Fig. 6, E to H) on par with the serum obtained from PCV13-immunized mice. The opsonophagocytic activity of PCV13-immunized mouse sera against nonvaccine serotypes likely reflects cross-reactive antibody responses and complement-mediated killing, as previously reported (*22*, *63*, *73*). The lower killing observed for serotype 33F is consistent with serotype-dependent variability in pneumococcal susceptibility to immune clearance, influenced by differences in capsule structure, complement interactions, and antigen accessibility (*74*, *75*). Together, these findings underscore the unique ability of ZPY-CpG-Ch–induced antibodies to mediate effective opsonophagocytic activity and bacterial clearance.

Our results align with earlier studies highlighting the critical role of opsonophagocytic antibodies in protecting against pneumococcal infection (*76*, *77*), specifically noting that opsonization enhances immune clearance in pneumococcal diseases. However, in contrast to antibody responses elicited by traditional conjugate vaccines (like PCV13), which are serotype-specific, the antibody response–induced ZPY-CpG-Ch formulation shows broader coverage, critically including nonvaccine serotypes. This enhanced breadth of coverage suggests that ZPY-CpG-Ch could complement existing vaccines by providing broad cross-serotype protection—a key advantage for global pneumococcal immunization efforts.

In parallel with the passive immunization experiments, we assessed whether vaccination with ZPY-CpG-Ch influenced nasopharyngeal colonization. Quantification of bacterial loads recovered from the murine nasopharyngeal tissue showed that ZPY-CpG-Ch did not significantly reduce pneumococcal carriage compared with control groups (fig. S16). These findings indicate that, under the conditions tested, the formulation elicited robust systemic antibody-mediated protection in invasive disease models while having a minimal effect on bacterial persistence in the nasopharynx. While reduction of colonization is desirable, protection against invasive disease remains a clinically meaningful end point. ZPY-CpG-Ch provides broad, serotype-independent protection and could be developed either as a stand-alone vaccine or in combination with conjugate vaccines to achieve both carriage reduction and disease protection.

### ZPY-CpG-Ch vaccination induces IL-17A–producing CD4^+^ T cell responses

**Fig. 7.**
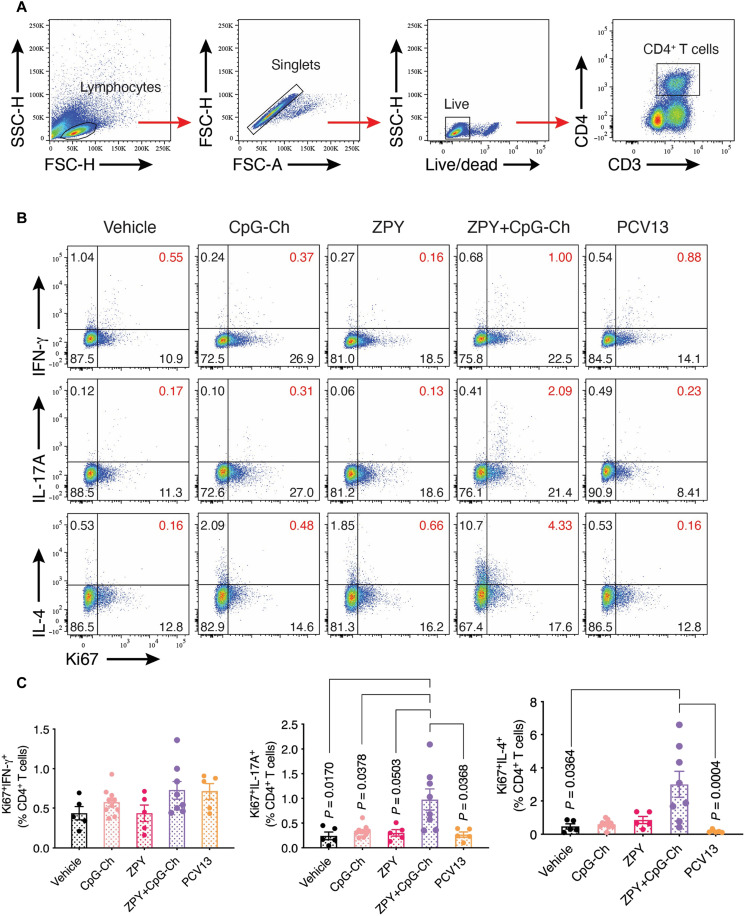
ZPY-CpG-Ch vaccination induces IL-17A–producing CD4^+^ T cell responses. Adult mice were immunized with either PBS (vehicle, *n* = 5), CpG-Ch (*n* = 10), ZPY (*n* = 5), ZPY adjuvanted with CpG-Ch (ZPY-CpG-Ch, *n* = 8), or PCV13 (*n* = 5). Lymphocytes were separated from the MLNs of immunized animals and analyzed for T cell activation. (**A**) Gating of live CD4^+^ T cells. (**B**) Representative flow cytometry plots for IFN-γ, IL-17A, and IL-4 expression in Ki67^+^CD4^+^ T cells. The frequencies of these T cell subsets were quantified as shown in (**C**). The Kruskal-Wallis test with Dunn’s multiple comparisons post test was used to test for statistical significance.

Vaccination with ZPY formulated with CpG-Ch promoted a significant increase in IL-17A–producing CD4^+^ T cells within MLNs of immunized mice. Single-cell suspensions from MLNs of mice immunized with either vehicle, ZPY alone, CpG-Ch alone, ZPY formulated with CpG-Ch, or PCV13 were stained and analyzed for CD3^+^CD4^+^ T cell subsets expressing IL-17A, IL-4, and IFN-γ (Fig. 7A, gating strategy). While the frequency of IFN-γ–positive T cells in the ZPY-CpG-Ch group was not found to be significantly different, the frequency of IL-4–positive T cells was found to be fourfold higher than that of PCV13-immunized mice. We also determined that the frequency of IL-17A–positive T cells in the ZPY-CpG-Ch group was significantly higher than in control groups (vehicle and ZPY alone), as illustrated in dot plots (Fig. 7B) and quantified in Fig. 7C, with the means ± standard error of mean (SEM) values indicating robust IL-17A cytokine responses. Our results align with findings from previous studies on pneumococcal protein vaccines, which indicate that IL-17A–producing CD4^+^ T cells play a central role in mucosal immunity against *S. pneumoniae* (*78*–*80*).

## DISCUSSION

The present study illustrates how a pangenome reverse vaccinology approach can be used to identify highly conserved protein antigens with the potential to provide cross-serotype protection against *S. pneumoniae*. Rather than pursuing ever-increasing capsular valency, our strategy focused on conserved, surface-exposed proteins that remain immunologically accessible across pneumococcal lineages. This direction is consistent with earlier studies indicating that vaccines targeting conserved proteins may circumvent the limitations of polysaccharide-based approaches (*81*, *82*) and help address the challenge of serotype replacement observed after widespread vaccine use (*83*, *84*). While the current pipeline prioritized surface-exposed and secreted proteins to maximize antibody accessibility, protective immunity against *S. pneumoniae* is not exclusively antibody-dependent. Intracellular and membrane-associated proteins may also contribute via T cell–mediated responses. Although not prioritized here, these antigen classes may be incorporated in future iterations to further broaden immune coverage.

Our antigen selection pipeline was designed to balance the breadth of coverage with biological plausibility. By focusing on proteins predicted to be surface associated or secreted and filtering out those with homology to human sequences, we ensured that candidates were both accessible to immune recognition and unlikely to elicit autoimmune responses. The conserved nature of these selected proteins across geographically and temporally diverse isolates suggests that they are functionally constrained and less prone to antigenic variation—an important feature for maintaining efficacy across global pneumococcal populations (*85*). This strategy addresses one of the long-standing challenges in pneumococcal vaccine development, i.e., the capacity of the pathogen for genetic plasticity and immune evasion (*86*, *87*).

Among the final shortlisted antigens, three proteins—ZmpB, PavA, and a YfhO-*like* protein—emerged as strong candidates on the basis of their predicted accessibility, conservation, and functional relevance. The selection of these three antigens reflects those that consistently scored highly across these criteria and represents a pragmatic balance between antigenic breadth and feasibility for recombinant production and downstream translational development. ZmpB, a zinc metalloprotease, has been previously implicated in pneumococcal virulence and modulation of host immune responses (*51*, *52*, *88*), which aligns with its immunogenic potential observed in our study. PavA is involved in nutrient acquisition and adhesion to host cells and is also a well-established virulence factor (*54*, *89*), underscoring its potential as a vaccine candidate. The presence of these known virulence factors in our final selection confirms the robustness of our pipeline and is consistent with previous findings where virulence-associated proteins serve as effective vaccine targets. In *S. pneumoniae*, the *yfhO* gene encodes a conserved membrane protein whose function remains largely uncharacterized; to date, the roles of YfhO-*like* genes have not been reported in this bacterium. However, on the basis of sequence homology and domain architecture, *YfhO* is annotated as a putative glycosyltransferase of the PMT (protein *O*-mannosyltransferase) family, suggesting a potential role in protein O-mannosylation. Members of the YfhO family, found across diverse bacterial species, typically contain 8 to 13 transmembrane helices and a conserved DXD motif, features characteristic of the GT-C fold glycosyltransferases, which facilitate the transfer of mannose from lipid-linked donors to serine or threonine residues on proteins. In related organisms, such as *Listeria monocytogenes* and *Bacillus subtilis*, YfhO homologs have been implicated in cell surface modification processes and are regulated by two-component systems such as YvrGHb (*90*, *91*). Extrapolating from these models, YfhO in *S. pneumoniae* may play a similar role in modifying surface or secreted proteins, potentially contributing to virulence, immune evasion, or adhesion. Elucidating its function could reveal new aspects of pneumococcal protein glycosylation and provide insights into uncharacterized pathways critical for *S. pneumoniae* pathogenesis.

Our immunological assays revealed varying IgG antibody responses to these antigens across different demographic and clinical groups, including children, healthy adults, patients with CAP, HIV-positive adults, and elderly individuals. Higher IgG levels in children compared to elderly adults suggest age-related variations in immune response to *S. pneumoniae* antigens, potentially due to immunosenescence, which is known to diminish immune responses in older adults (*92*). This age-related disparity in immunity highlights a need for vaccines that can elicit stronger immune responses across all age groups, particularly given the vulnerability of both the young and the elderly to pneumococcal infections (*93*).

In murine models, immunization with ZPY combined with CpG-Ch demonstrated dose-dependent immunogenicity. The CpG-Ch adjuvant elicited a robust antigen-specific IgG response, consistent with a prior study that has shown CpG oligonucleotides to be effective immune stimulants, particularly in inducing T_H_1-type responses (*60*). This enhanced immune activation is critical for combating bacterial infections, as it promotes humoral and cellular immunity, providing a broader and more durable immune response compared to Alum, which primarily elicits antibody-mediated immunity. Notably, the ZPY-CpG–adjuvanted formulation yielded higher survival rates and reduced bacterial load compared to the Alum-adjuvanted formulation, aligning with previous findings that CpG-based adjuvants can enhance bacterial clearance (*94*). ZPY-CpG-Ch was noninferior to the commercially available PCV13 in terms of protective efficacy against pneumococcal pneumonia in both adult and neonatal mice. The efficacy of ZPY-CpG-Ch across different age groups indicates its potential as a universal vaccine candidate, providing broader protection in diverse populations. Moreover, the observed cross-protection against non-PCV13 serotypes, including serotypes 8, 11A, and 33F, further validates the potential of ZPY-CpG-Ch to address gaps in existing pneumococcal vaccination strategies. These non-PCV13 serotypes are associated with severe disease outcomes, particularly in regions where nonvaccine serotypes are prevalent (*69*, *95*, *96*).

The passive immunization studies, in which sera obtained from ZPY-CpG-Ch–immunized mice conferred protection in naïve mice, underscore the functional efficacy of ZPY-induced antibodies. Our findings indicate that anti-ZPY antibodies not only recognize pneumococcal antigens but also mediate bacterial clearance and enhance survival, supporting the role of these antibodies in immune protection. The use of in vitro opsonophagocytic assays further confirmed the functional capability of ZPY-CpG-Ch–induced antibodies to promote phagocytosis and clearance of pneumococci, reinforcing the potential of our vaccine formulation in preventing invasive disease. We also generated data indicating that, under the conditions tested, ZPY-CpG-Ch does not significantly reduce the nasopharyngeal carriage of pneumococcus. While reducing colonization has been a traditional goal for pneumococcal vaccines, as it helps lower transmission and create herd immunity, it may not be essential for effective disease prevention (*97*). Nasopharyngeal colonization is part of the upper respiratory microbiome balance, and disrupting this through vaccination could have unpredictable long-term effects. This perspective is shared among researchers who believe that focusing strictly on eliminating carriage has held back broader vaccine development, especially protein-based pneumococcal vaccines (*97*, *98*). Instead, the emphasis might shift toward disease prevention rather than complete carriage elimination. In light of these findings, we are exploring the possibility of coformulating ZPY with existing conjugate vaccines. This combined approach may offer robust protection against disease without disrupting the microbiome in the upper respiratory tract.

This study has some limitations that should be considered when interpreting the findings. First, protective efficacy was assessed at a single time point corresponding to peak immune responses following immunization, and the durability of protection was not evaluated. Assessment of long-term immunity, including the persistence of both antibody and cellular responses, will be an important next step to determine the potential of our vaccine platform for sustained protection. Second, while ZPY-CpG-Ch conferred robust protection against invasive disease, it did not significantly reduce pneumococcal colonization under the conditions tested. This likely reflects differences in the immune mechanisms required for systemic protection versus mucosal immunity. In particular, effective prevention of colonization is thought to depend on mucosal immunity, including secretory IgA responses and tissue-resident immune cells, which may not be optimally induced by intramuscular immunization. In contrast, the observed protection against invasive disease is consistent with the induction of systemic IgG and T_H_1/T_H_17-mediated responses. Together, these findings highlight that distinct immunological pathways underlie protection against disease and colonization. Licensed pneumococcal conjugate vaccines such as PCV13 have been shown to reduce the carriage of vaccine-type serotypes, but this effect is largely serotype-specific, contributing to serotype replacement (*99*, *100*). A further consideration relates to adjuvant selection. While CpG-Ch was used here because of its capacity to promote T_H_1/T_H_17–skewed responses, it represents only one strategy within a broader and evolving landscape of adjuvants for protein-based pneumococcal vaccines. Alternative adjuvants may differentially enhance mucosal immunity, antigen presentation, or tissue-resident immune responses, potentially improving outcomes relevant to colonization. Accordingly, future studies—particularly those focused on preventing nasopharyngeal carriage—should explore additional adjuvant systems alongside alternative immunization strategies, such as mucosal delivery routes or combination approaches, to optimize local and serotype-independent protection.

In conclusion, the data presented in this study provide compelling evidence for the efficacy of ZPY-CpG-Ch as a universal pneumococcal vaccine candidate. Through a rigorous unbiased computation-driven antigen selection process, followed by immunological validation in both human sera and clinically relevant animal invasive disease models, we identified a pool of conserved antigens that have the potential to elicit broad and protective immune responses against diverse pneumococcal strains. ZPY-based vaccine–adjuvanted formulations could serve as stand-alone immunization or complement existing vaccines, offering enhanced immunity against pneumococcal disease across diverse populations and serotypes. Our future studies will focus on further preclinical and clinical evaluation of ZPY-based vaccine formulation, including its breadth of coverage across all known serotypes with a particular emphasis on serotype 3 (*101*). Together, our work contributes to the growing body of evidence supporting protein-based vaccines as viable alternatives to traditional conjugate vaccines, with the potential to broaden protection and mitigate the impact of serotype replacement.

## MATERIALS AND METHODS

### Large-scale comparative genomic analysis

An initial dataset of 3400 pneumococcal isolates from the Pneumococcal Genome Library (*102*) was used in investigations of the core genome content. We studied the core genome gene complement—as described in the workflow outlined in Fig. 1. We subsequently expanded our genomic analysis to the entire dataset (20,980) composed of the pubMLST Pneumococcal Genome Library and of all the Genbank pneumococcal isolates (accession date: 15 April 2024).

#### Quality control

To examine the pneumococcal genome population, a core SNP analysis was carried out using Snippy 4 with -ctgs parameter. Because of the large memory requirement in the alignment concatenation, genomes were run through the pipeline in batches of 4000 and the full alignments concatenated. The snp-sites program was subsequently executed on the concatenated alignment. FastBAPS (*41*) was then used in hierarchical clustering with the optimize prior setting as “baps.” From each cluster, a best representative genome was identified using (i) the assembly statistics of each genome assembly (N50, number of contigs, and total length) and (ii) the BUSCO results against the *Lactobacillales* dataset to analyze assembly completeness and cleanness. For the scale-up to >20,000 genomes, a mash triangle was initially run across all the collected genome assemblies (20,980). A plot was created from the mash results (not shown) using the QC_cmds pipeline from Panaroo (*103*), from which cutoffs to identify outliers were chosen as values between 0.01 and −0.01, where outliers were defined as results less than or greater than those values. All genomes inside the cutoff were automatically assigned 100 for completeness, while 519 outlier assemblies were subjected to a full BUSCO run (*104*). A total of 22 assemblies were then discarded as poorer quality, and snippy was run with fastBAPS as before. The best representative genome per hierarchical cluster was therefore chosen as the assembly with the highest completeness and cleanness as determined by the BUSCO score and additionally that with the highest N50 value, lowest number of contigs, and longest genome length. These best representative genomes from each cluster were used to identify the core protein set. Where protein sequences were examined directly for sequence conservation, all predicted proteins from the final genome set of 20,958 assemblies were included.

#### Workflow use

All genome assemblies were subjected to gene prediction with Prodigal version 2.6.3 (*105*), and protein FASTA files were generated for each strain. The computational workflow outlined in Fig. 1 was applied to the complete dataset. The computational pipeline comprised three main stages: data curation, antigen screening, and candidate prioritization. Genome assemblies were first curated and analyzed following assembly (SPAdes) (*106*) and annotation (Prokka) (*107*). During scale-up to the whole dataset of genomes, quality control was carried out as an initial step, plotting pairwise MASH distance with the method for Panaroo quality control. A cutoff was identified (−0.01, 0.01), and BUSCO was run on all the outliers, discarding genomes less than complete. Core genome analysis was conducted using Panaroo (*103*) to identify genes conserved across the dataset, enabling prioritization of antigens with minimal sequence variability and broad population coverage. Candidate proteins were subsequently filtered using a multiparameter approach. Subcellular localization was predicted using PSORTb 3.0 (*108*) to enrich for surface-exposed or extracellular proteins. Signal peptides and lipoproteins were identified using SignalP5.0 (*109*), and membrane topology was assessed using DeepTMHMM (*110*) to exclude multipass transmembrane proteins. Antigenicity was evaluated using Vaxign (*111*), and homology to the human proteome was evaluated using BLASTp. Candidates were scored on the basis of these criteria, and high-ranking proteins were shortlisted. Shortlisted antigens were further evaluated for physicochemical stability using ProtParam (*112*) and for structural properties using AlphaFold (*113*) to assess the folding and accessibility of predicted antigenic regions. We additionally performed structural similarity analysis using Foldseek (*114*), which did not identify significant similarity beyond pneumococcal proteins, further supporting the specificity of the selected pneumococcal antigens. The resulting data were analyzed to identify the most promising candidates, and the prevalence of the top three antigens was assessed across the complete dataset.

### Recombinant protein production

The expression, purification, and endotoxin removal of all recombinant pneumococcal proteins used in this study were carried out by the University of Liverpool GeneMill Core Facility. *Escherichia coli*–optimized synthetic genes and their encoding proteins for each of the vaccine candidates, namely pneumococcal adherence virulence factor A (PavA, protein ID: 445928848), a YfhO-*like* transmembrane protein (YfhO-*like*, protein ID: 446757434), and zinc metalloprotease B (ZmpB, protein ID: 446395105), were obtained from GeneArt (Thermo Fisher Scientific, UK). For each vaccine antigen, 6xHis-tagged C-terminal recombinant pneumococcal proteins were cloned into *E. coli* BL21 (DE3) cultured in Terrific Broth (Thermo Fisher Scientific). On the basis of DeepTMHMM analysis (*110*), we expressed the extracellular region of the multipass YfhO-*like* protein by amplifying and subcloning the Met^435^-Gly^790^ segment (∼40 kDa) as a T7-driven, SUMO-tagged construct. PavA was expressed in its full length, i.e., from Met^1^ to Ser^551^ (∼63 kDa) as a T7 promoter C-term 6xHis tag recombinant protein. The extracellular domain of ZmpB, as identified by the DeepTMHMM server, which comprises two peptidase domains (N- and C-), i.e., Met^140^ to Ala^1876^, ∼148 kDa, was amplified and subcloned as a T7 promoter C-term 6xHis tag truncated protein. All 6xHis-tagged proteins were recovered from HiPrep IMAC FF columns (Cytiva Life Sciences) and passed four times through Pierce High-capacity endotoxin removal spin columns (Thermo Fisher Scientific). Residual endotoxin levels were confirmed to be less than 0.05 units/ml using a Pierce Chromogenic Endotoxin Quant Kit (Thermo Fisher Scientific). Throughout this manuscript, the term ZPY is used to denote the combined formulation of ZmpB, PavA, and Y*fhO*-like proteins.

### Bacterial isolates

All pneumococcal strains used in the study were clinical isolates obtained from biobank of isolates archived at the Liverpool Royal Hospital (RLBUHT) or at the Malawi Wellcome Trust Centre, Blantyre, Malawi (table S1). Pneumococcal identification was confirmed by an optochin sensitivity test, and serotypes were determined using the standard Quellung reaction with pneumococcal capsule–specific antisera (Statens Serum Institute, Copenhagen, Denmark). All isolates were stored at −80°C on Protect cryobeads (Pro-Diagnotics Lab, UK) and subcultured on blood agar base (Oxoid, UK) plates supplemented with 5% (v/v) sterile defibrinated horse blood (TCS Biosciences, UK) at 37°C under anaerobic conditions for 18 hours. Single colonies were grown in brain heart infusion broth (Oxoid, UK) supplemented with 20% (v/v) fetal bovine serum until the exponential phase. Bacterial suspensions were aliquoted and stored at −80°C. Multilocus sequence typing (MLST) was also carried out on all isolates.

### Ethics statement and animals

All animal experiments were carried out in accordance with UK Animals (Scientific Procedures Act) 1986 and the care and maintenance guidelines of the University of Liverpool. All animal protocols were approved by the University of Liverpool Animal Welfare and Ethics Committees and under UK Home Office Project Licence PB6DE83DA. Wild-type C57/BL6J female mice were obtained from Charles River Laboratories (Margate, UK) at 6 to 8 weeks old (adult immunization studies) or at G12 ½ days of gestation (neonate immunization studies).

### Mouse challenge studies in pneumonia models

Age-matched (6 to 8 weeks) C57BL/6 female mice (Charles River, Margate, UK) were used for pneumonia and sepsis models. Inocula from pneumococcal cultures were administered to mice in a sterile phosphate-buffered saline (PBS) vehicle solution at varying densities: In the pneumonia model, mice were anesthetized with a mixture of O_2_ (oxygen gas) and isoflurane and infected intranasally with 5 × 10^5^ CFU/50 μl per mouse of a hypervirulent *S. pneumoniae* strain. Mice were periodically scored for clinical signs of disease and humanely euthanized by carbon dioxide (CO_2_) inhalation followed by cervical dislocation—in accordance with UK regulatory and institutional ethical guidelines—when they became moderately lethargic at predetermined times postinfection based on Morton’s scheme (table S2). All experiments were conducted over a period of up to 72 hours of monitoring.

### Reagents

Protasan ultrapure chitosan chloride (CL213; endotoxin ≤100 EU/g) was obtained from NovaMatrix. Unmethylated cytosine-phosphorothioate-guanine oligodeoxynucleotide (CpG ODN 1826, class B/group 2) was supplied by Oligos Etc. (US). The term CpG-Ch is used throughout the manuscript to denote the combined formulation of CpG (10 μg per dose) and Protasan chitosan salt (500 μg per dose). Flow cytometry antibodies and ELISA kits were obtained from BD, eBioscience, or BioLegend, as listed in table S3. Alhydrogel (referred to as Alum throughout this manuscript) was procured from Brenntag Biosector A/S (now part of Croda International Plc, UK). PCV13 was purchased from Kays Medical Supplies, UK.

### Immunization protocol

In murine adult studies, pneumococcal candidate antigen formulations were administered to 6- to 8-week-old female C57BL/6 mice (*n* = 10 per group, unless stated otherwise) by three sets of 50-μl intramuscular immunizations (prime, boost 1, and boost 2) at weeks 0, 2, and 4, i.e., three doses at a 2-week interval, over a period of 28 days. In murine pup studies, a 10-μl volume of the solution was administered intramuscularly to C57BL/6 neonatal mice (*n* = 5 to 8 per group) on postnatal days 7 (prime), 14 (boost 1), and 21 (boost 2). Control groups were administered with either PBS (vehicle), adjuvant only (CpG-Ch or Alum), or pneumococcal antigen(s)–only solution (i.e., ZPY combined or single antigen formulations), and results are presented in relevant figures to establish baseline responses. Because of experimental constraints, not all control groups were included in each comparative experiment. In addition to the negative placebo control group, our vaccine formulations were evaluated against the marketed pneumococcal vaccine PCV13 (tradename Prevnar-13). Each antigen was administered at a dose of 1.0 μg per immunization. For the trivalent ZPY formulation, this corresponded to a total protein dose of 3.0 μg (1.0 μg per antigen), ensuring equivalent per-antigen dosing across all experimental groups.

### Passive immunity transfer studies

Adult mice (6- to 8-week-old female C57BL/6) previously immunized with either vehicle or ZPY antigen formulation adjuvanted with CpG-Ch (ZPY-CpG-Ch) were culled by cardiac puncture, and sera (100 to 150 μl per mouse) were transferred into naive recipient mice via intravenous administration. At 4 hours posttransfer, animals were challenged intranasally (pneumonia model) with hypervirulent serotype 1 *S. pneumoniae* (*115*). Mice survival, pain scores, and viable CFU counts in blood and lung samples were recorded.

### Tissue samples for counting bacterial viable counts

Whole blood and lung tissues were collected freshly according to disease progression following pneumococcal challenge and following the UK Animals Scientific Procedures Act. Blood was taken by cardiac puncture. Lung lobe samples were dissociated by an electronic tissue homogenizer (T10 Ultra Turrax, IKA, Scientific Labs Ltd., UK). Viable pneumococcus counts recovered from lung homogenates and blood samples were determined according to the Miles and Misra method at the indicated intervals after pneumococcal infection by 10-fold serial dilution in sterile PBS and plated on blood agar containing 5% (v/v) defibrinated horse blood (TCS Biosciences, UK) and gentamicin (10 μg/ml; Sigma-Aldrich). Once dry, plates were incubated in 5% (v/v) CO_2_ at 37°C overnight, and bacterial colony numbers were counted on the following day.

### Ex vivo analysis of vaccine-induced immunity

To characterize the antipneumococcal adaptive immunity induced by active immunization, ZPY-specific IgG antibody titers and IgG subclasses (IgG1 and IgG2c) were analyzed by indirect ELISA assays. Mouse immune sera were prepared from blood samples collected by cardiac puncture 1 week post–tertiary vaccination (boost 2) (Fig. 4A). High-binding microtiter plates (Corning) were coated with pneumococcal protein candidates (1 μg/ml; ZPY) in carbonate-bicarbonate buffered saline (50 mM, pH 9.2) and incubated at 4°C overnight. Sera were serially diluted by twofold starting at 1:50 in diluent buffer containing 0.05% Tween 20 and 0.1% bovine serum albumin (BSA) in PBS. The levels of pneumococcal antigen–specific IgG and IgG subclasses were given semiquantitatively on the basis of OD values (OD units at 450 nm). All biotin-conjugated antibodies were obtained from Southern Biotech (US) via their distributor 2BScientific, UK, while the extravidin-enzymatic conjugate was purchased from Sigma-Aldrich UK (E2886). For cell-mediated immunity analyses, single-cell suspensions were prepared from MLNs, as described elsewhere (*116*). MLNs were mechanically dissociated by pushing through a 70-μm cell strainer to generate a single-cell suspension and restimulated ex vivo in the presence or absence of ZPY pneumococcal antigens before flow cytometry staining.

### OPK assay

OPK assays were performed to test the ability of vaccinated mouse antiserum to opsonize viable pneumococci and mediate killing. A reference OPK assay described elsewhere (*117*) was used with minor modifications. A reaction mixture consisting of heat-inactivated serum obtained from either immunized or nonimmunized mice, differentiated HL-60 cells, pneumococci, and baby rabbit complement was prepared. Briefly, 5 × 10^4^ dimethylformamide-differentiated HL-60 cells were incubated with 5 × 10^2^ opsonized *S. pneumoniae* (ratio, 100:1) and baby rabbit complement for 45 min at 37°C and 5% CO_2_. Human normal immunoglobulin—IVIG (Gamunex 10%, Grifols, UK)—was used as the positive control for antibody-mediated killing (at a final dilution of 1:16) and as a source for pneumococcus-specific antibodies. Reaction mixtures were plated on blood agar plates, and viable colony counts were performed after ∼18 hours of incubation period under anaerobic conditions at 37°C.

### Protein-specific antibody titrations in children and healthy adult sera

The levels of specific IgG antibodies to ZmpB, PavA, and YfhO-*like* antigens were determined by ELISA in acellular sera (*n* = 31 in total) obtained from *n* = 13 children (aged between 2 and 9 years), *n* = 5 healthy adults (aged 18 to 45 years), *n* = 5 patients presenting pneumococcal CAP, *n* = 5 adults living with HIV, and *n* = 3 elderly patients aged 65 to 75 years of age. High-binding microtiter plates (Corning UK) were coated with total protein (1 μg/ml; either as single YfhO-*like*, PavA, or ZmpB protein or as a trivalent combination, coined ZPY) in carbonate-bicarbonate buffered saline (50 mM, pH 9.2) and incubated at 4°C overnight. Blocking was done with 1% (w/v) BSA in PBS. Sera were serially diluted by twofold starting at 1:20 in 0.5% (w/v) BSA in PBS. Alkaline phosphatase–conjugated monoclonal anti-human IgG (Sigma-Aldrich UK, A2064-1ML) was used as the secondary antibody. An OD of ≥0.04 (two standard deviations from the control serum) for all measurements was considered positive. Samples with undetectable IgG levels to ZmpB, YfhO-*like*, and/or PavA were assigned a value equivalent to half the detection limit. The protein-specific IgG results are given semiquantitatively on the basis of OD values (OD units at 405 nm). These were calculated from the mean OD readings of triplicate samples after subtraction of the OD readings of serum-negative control wells.

### Flow cytometry

Single cells from mouse lung and MLN were incubated with Live/Dead Fixable Aqua Stain (Thermo Fisher Scientific, UK) at 1:1000 to determine viable cells. Cell surface staining was performed with fluorochrome-conjugated anti-mouse monoclonal antibodies (table S3) in the presence of True-Stain Monocyte Blocker (1:20) (BioLegend, UK). Cell-mediated immunity was determined by measuring pneumococcal antigen–specific CD3^+^CD4^+^ T cell responses. For intracellular cytokine detection, brefeldin A (5 μg/ml; Sigma-Aldrich UK) was included before staining in the last 6 hours of cell culture stimulation at 37°C and 5% CO_2_. Cells were fixed with Fixation Buffer (BioLegend UK) and then washed with Intracellular Staining Permeabilization Wash Buffer (BioLegend). Permeabilized cells were then stained with monoclonal antibodies against intracellular cytokines (IL-4, IL-17, and IFN-γ) for T_H_1/T_H_2 and T_H_17 panels. Stained samples were acquired on a BD FACSCanto II and analyzed using FlowJo 10.4.

### Statistical analysis

All results were expressed as individual mouse data with the means ± SEM, unless otherwise stated. For the comparison between two groups, the nonparametric Mann-Whitney *U* test was used. For comparisons among three or more groups or experiments with multiple variables, the Kruskal-Wallis test with Dunn’s multiple-comparisons post test was applied. Survival curves were analyzed using the Kaplan-Meier method and compared with the log-rank (Mantel-Cox) test. For comparisons assessing noninferiority between vaccine groups (ZPY-CpG-Ch versus PCV13), analyses were conducted using this approach, with group sizes of *n* = 10 mice per experimental arm, unless otherwise stated*. P* ≤ 0.05 was considered statistically significant. Statistical analyses were performed using GraphPad Prism (version 10.0).
