# Supplementary material for: ZPY-CpG-Ch, a broad coverage vaccine against Streptococcus pneumoniae
Source: Sci Adv. 2026 Aug 7;12(32):eaee7981. doi: 10.1126/sciadv.aee7981 (PMC13450215; doi:10.1126/sciadv.aee7981)
Supplement: Supplementary file 1 [file sciadv.aee7981_sm.pdf]

Science Advances
AAAS

# Supplementary Materials for

## ZPY-CpG-Ch, a broad coverage vaccine against *Streptococcus pneumoniae*

Teerawit Audshasai *et al.*

Corresponding author: Marie O'Brien, marie.obrien@citystgeorges.ac.uk;
Aras Kadioglu, a.kadioglu@liverpool.ac.uk

*Sci. Adv.* **12**, eaee7981 (2026)
DOI: 10.1126/sciadv.aee7981

**This PDF file includes:**

Figs. S1 to S16
Tables S1 to S3

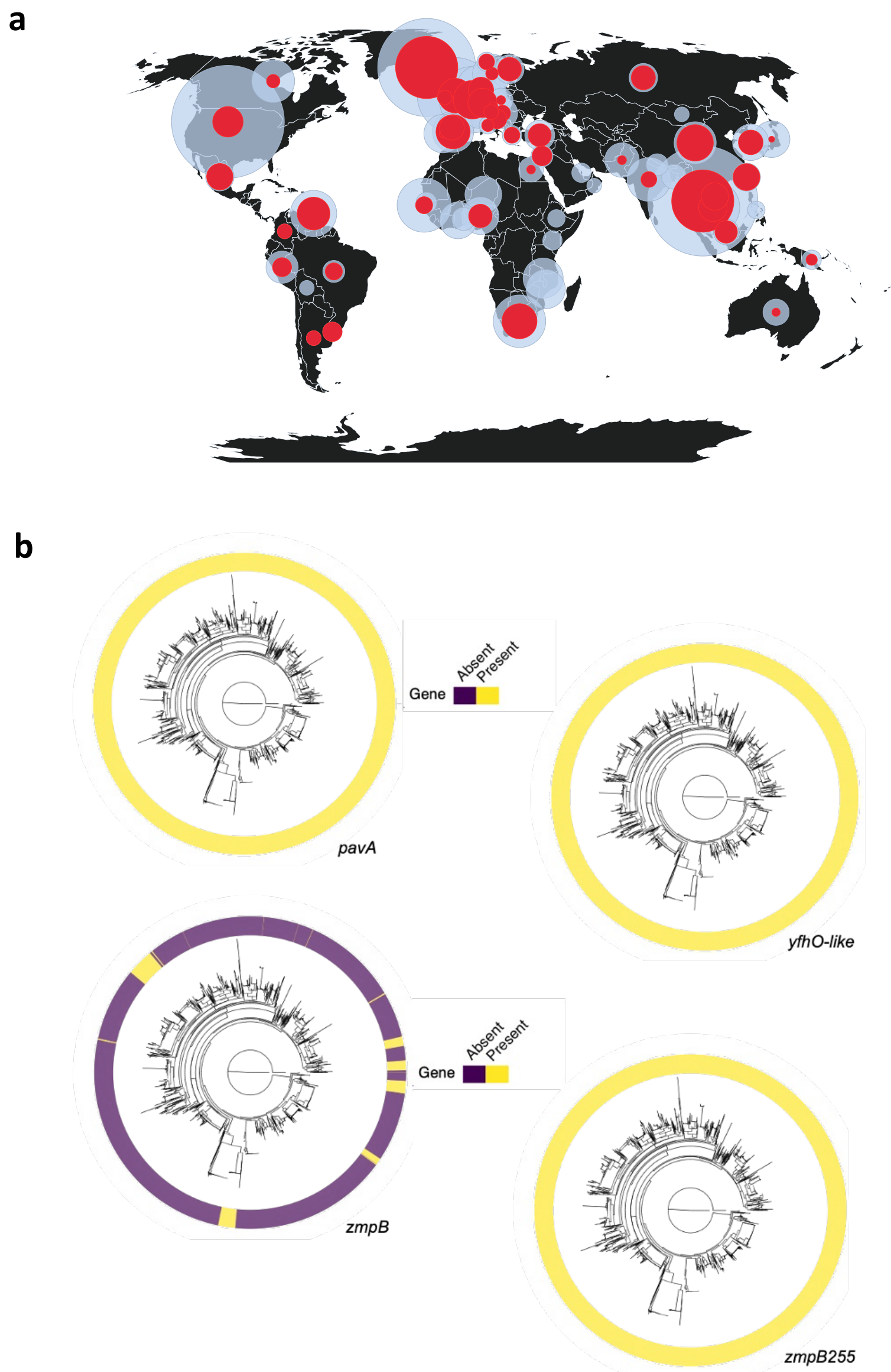


**Fig. S1. Geographical distributions of S. pneumoniae strains used in analytical pipeline & species-wide conservation of vaccine antigen candidates.** (a) World map plot visualising the distribution of serotypes. Circles were plotted on geographic coordinates from R Natural Earth package, using ggforce and geom circle together with the R cartogram Dorling library. The Dorling cartogram approach was applied which results in positional adjustments for clarity while maintaining proportional relationships. Radii of the circles were scaled to represent the proportions of different serotypes in various regions. For clarity in representing large variations across countries, the square root of the radius values was taken, followed by multiplication by 33 to enhance visual differentiation. (b) Analysis was performed on a randomised global dataset of 4,576 pneumococcal genomes from 21 countries across all continents except Australia. The visualisations depict gene presence and absence across the collection. pavA and the yfhO-like transporter gene are universally present in all isolates regardless of lineage or region, supporting their potential as broadly protective vaccine targets. zmpB, a non-conserved gene, is included to assess the value of incorporating variable antigens in vaccine design. Notably, while the N-terminal 255 aa region of zmpB is conserved across all isolates, the remainder of the gene shows extensive variability - highlighting that conventional assumptions about antigen variability may have caused promising candidates to be overlooked and warrant re-evaluation in future antigen discovery efforts.

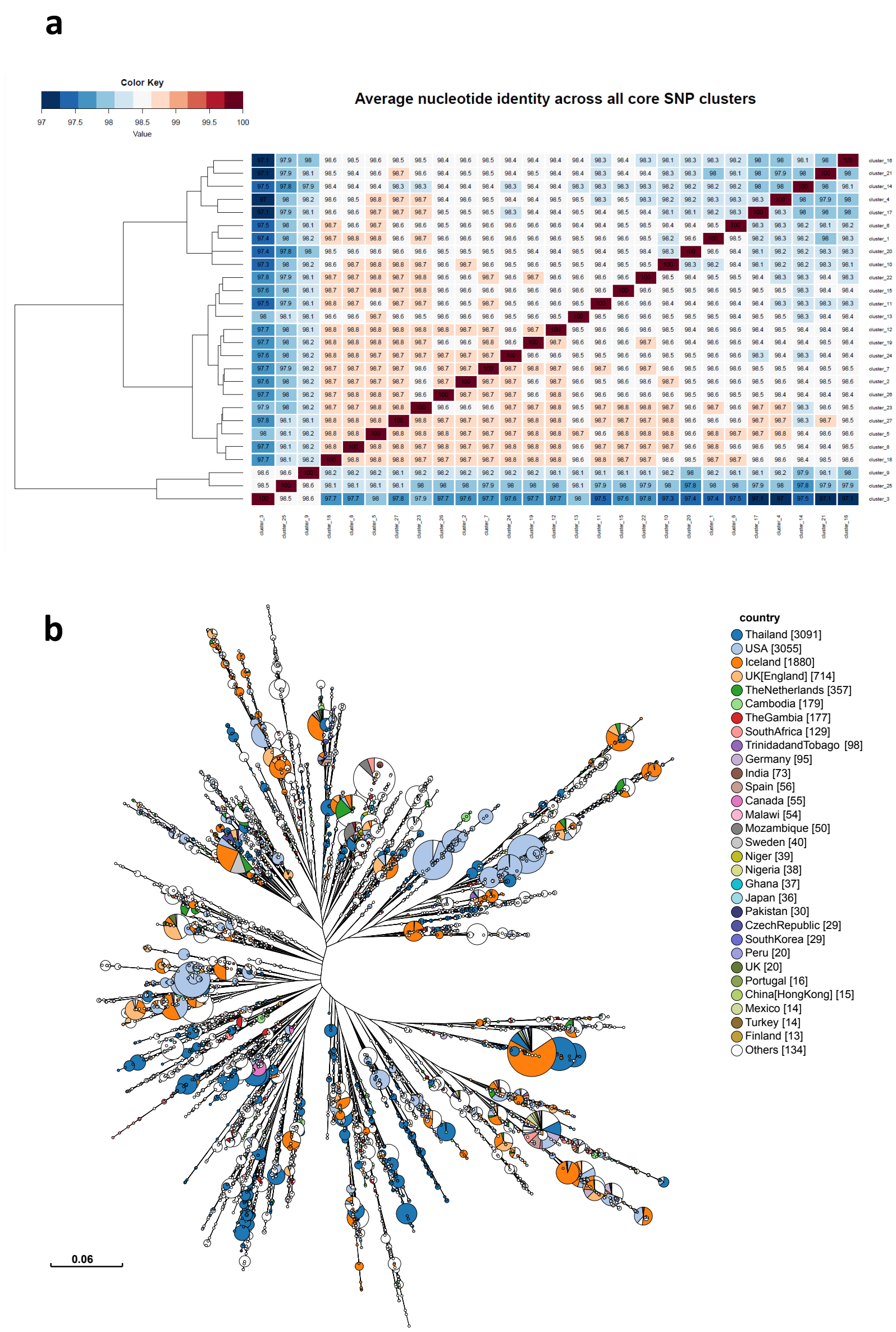


**Fig. S2. Average Nucleotide Identity (ANI) and SNP Minimum Spanning Tree.** (a) The core single nucleotide polymorphisms (SNPs) from all genomes were clustered using the FastBAPs Bayesian clustering approach, resulting in 34 distinct clusters. Despite the clustering, the genomes remain highly related. ANI values were calculated based on bidirectional BLAST best hits for all genes from a representative genome for each cluster (totalling 34 genomes). The selected representative genomes were chosen for their completeness (100% complete with no duplicate BUSCO genes) and optimized genome assembly (fewest contigs and largest genome size). The lowest ANI observed was 98%, indicating high genetic similarity. Red indicates the most closely related genomes, while darker blue denotes more distantly related genomes, with cluster 27 being the most divergent. (b) Whole Genome Core SNP Minimum Spanning Tree Coloured by Countries. This minimum spanning tree represents the genetic relationships among strains based on whole genome core SNP data. Each node corresponds to a unique genome, and the branches depict the evolutionary distances between them. The colours represent different countries, indicating the geographic origin of each strain. Closely related genomes form clusters, indicating geographical or epidemiological connections. Longer branches reflect more genetic divergence, suggesting potential geographic or evolutionary separation between strains from different regions.

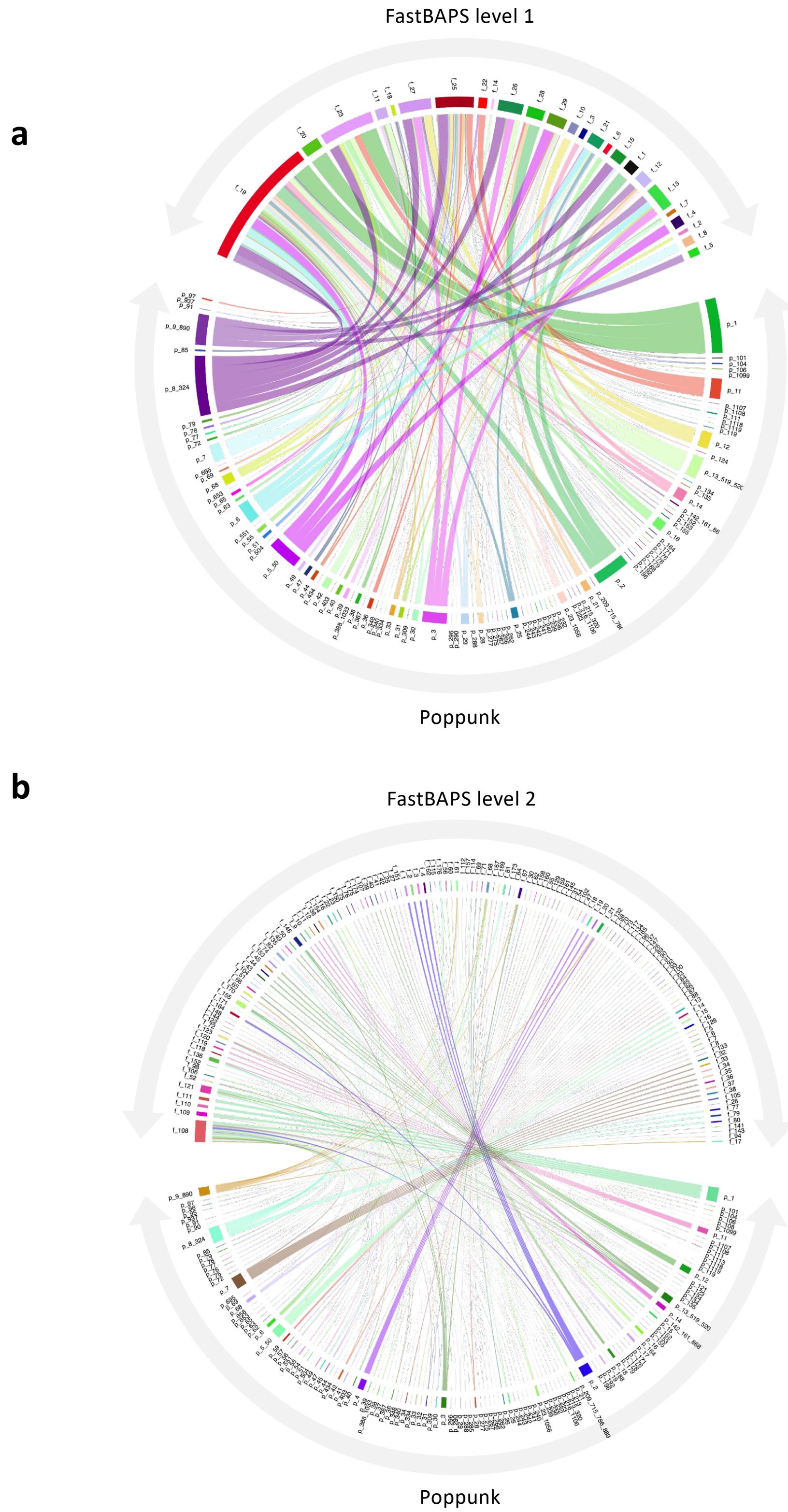


**Fig. S3. GPSC Poppunk Clusters Comparison and Analyses.** Figure showing a comparison of the fastBAPS hierarchical clusters with the PopPunk clusters. The fastBAPS is shown at the top of the figure with PopPunk clusters at the base. Panel (a) shows the comparison with fastBAPS level 1; panel (b) shows the comparison with fastBAPS level 2.

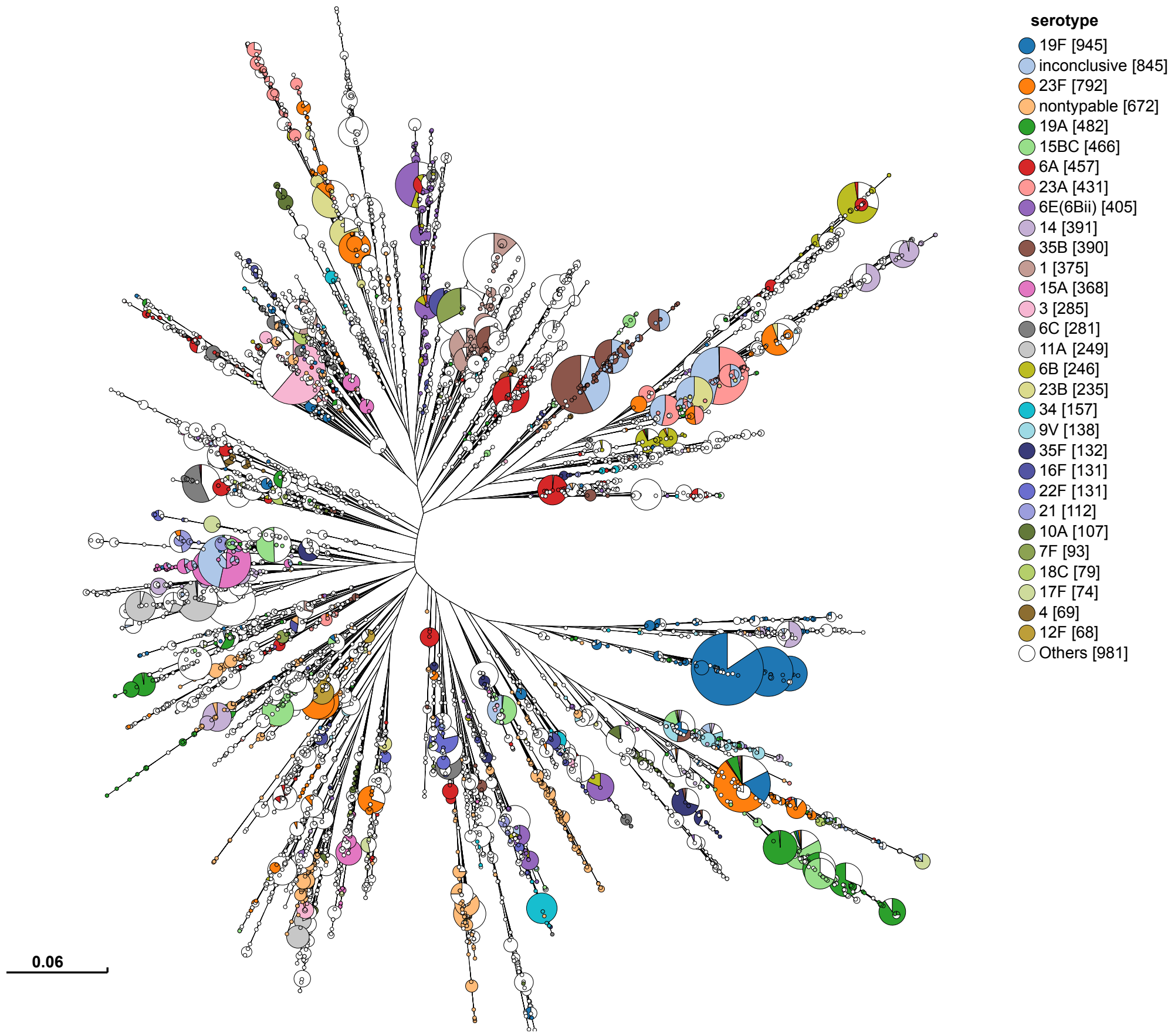


**Fig. S4. Minimum Spanning Tree (MST) Analysis of the Pneumococcal Genomes and Serotypes included in this study.** Figure showing the phylogenetic diversity where minimum spanning tree (MST) illustrates the relationships among the n=20,958 pneumococcal genomes used in this study. Each node represents a distinct cluster, and the colours indicate different serotypes. The size of the nodes corresponds to the number of isolates within each serotype, and the length of the branches between nodes reflects the genetic distance between the serotypes. Closely related serotypes are connected by shorter branches, while more distantly related serotypes have longer connections.

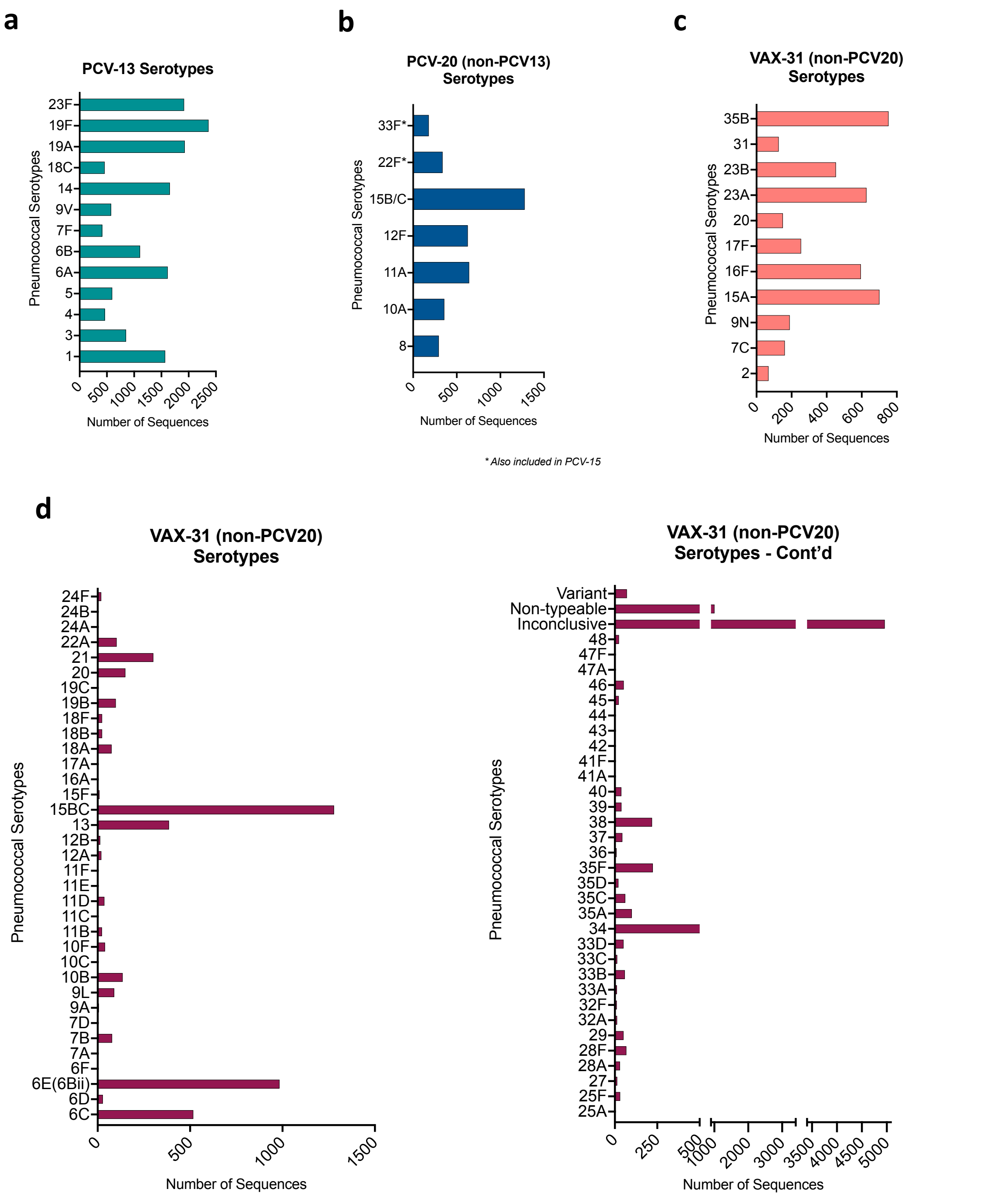


**Fig. S5. Frequency of Pneumococcal Genome Sequences in our Dataset, Grouped by Vaccine and Non-Vaccine Serotypes.** This figure displays the distribution (bar charts) of pneumococcal genome sequences used in the dataset, categorised by vaccine and non-vaccine serotypes. The vaccine serotypes are divided into groups based on the pneumococcal conjugate vaccines (PCV) they are covered by: PCV-13 (serotypes included in the 13-valent vaccine), PCV-15 and PCV-20 (serotypes only covered in the 15- and 20-valent vaccines, respectively), VAX-31 (serotypes only covered in a 31-valent formulation), and non-vaccine serotypes. The frequency of sequences from each group is represented, illustrating the relative prevalence of vaccine-covered vs. non-vaccine serotypes in the dataset.

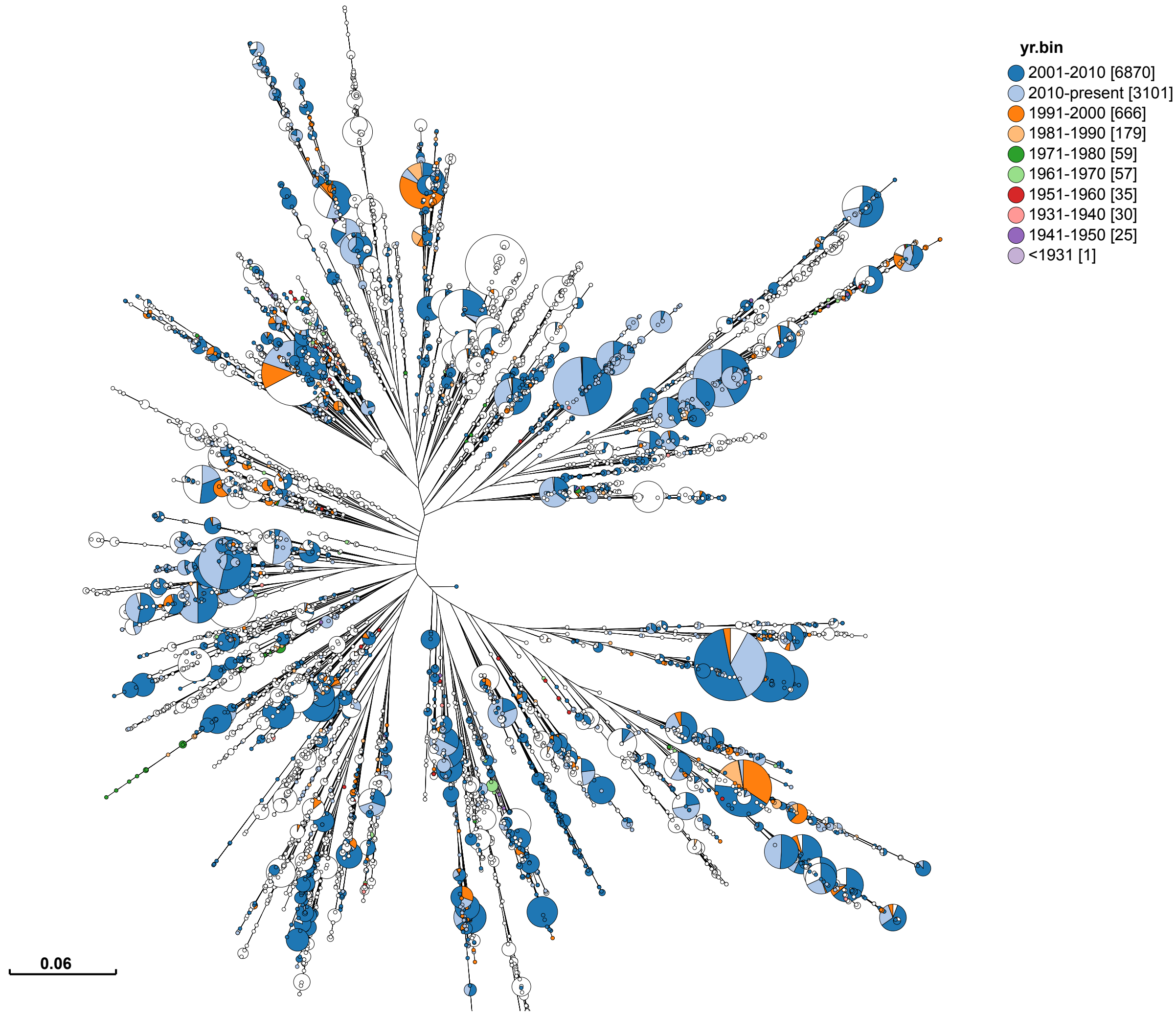


**Fig. S6. Minimum Spanning Tree (MST) Analysis of Pneumococcal Isolates Coloured by Year of Isolation.** This figure presents minimum spanning tree (MST) analyses of pneumococcal serotypes, illustrating their distribution over time. Pneumococcal genomes were grouped by decade, showing the evolutionary relationships among strains isolated in different time periods. The nodes, representing distinct clusters, are coloured according to their year of isolation, allowing for visualisation of genetic relatedness over time. Pre-2007 vs. post-2007 isolates, where 2007 marks the introduction of PCV13 into routine immunisation programmes, highlights the distribution of our dataset in the pre vs. post-PCV vaccination era. Shorter branches between nodes indicate closely related isolates, while longer branches represent more genetically distant serotypes. These analyses are useful for ensuring the broad coverage of ZPY-based formulations by considering the evolutionary dynamics of pneumococcal population structure over time, especially in response to vaccine-driven selection pressure.

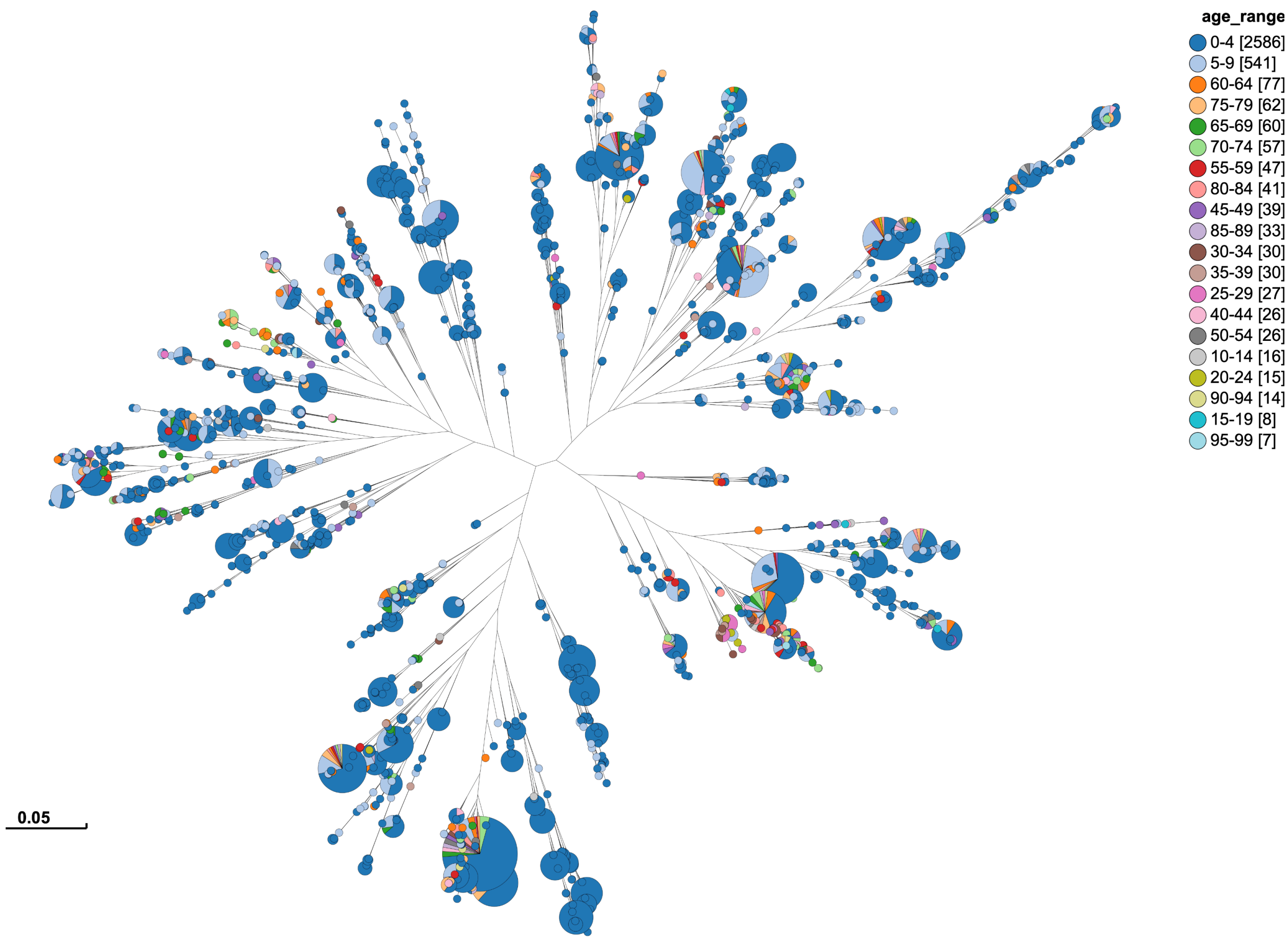


**Fig. S7. Minimum Spanning Tree (MST) Analysis of Pneumococcal Isolates Coloured by Age Group.** This figure displays a minimum spanning tree (MST) analysis of pneumococcal serotypes, organised by the patient age groups from which the bacterial isolates were obtained. Each node represents a distinct genetic cluster, with nodes coloured according to age categories (i.e., ranging from 0 to 99 years of age). The length of the branches connecting nodes reflects the genetic distance between clusters, with shorter branches indicating closer genetic relatedness. This analysis illustrates the phylogenetic distribution of the n=20,958 pneumococcal genome assemblies used in this study within different age groups, revealing potential age-specific patterns of genetic diversity. These analyses are useful for ensuring that ZPY-based vaccine formulation provides broad protection by accounting for the genetic diversity of pneumococcal strains across different patient groups. This approach allows the formulation to deliver comprehensive coverage, not only across various serotypes and over time but also across diverse age groups, ensuring a more universally effective vaccine.

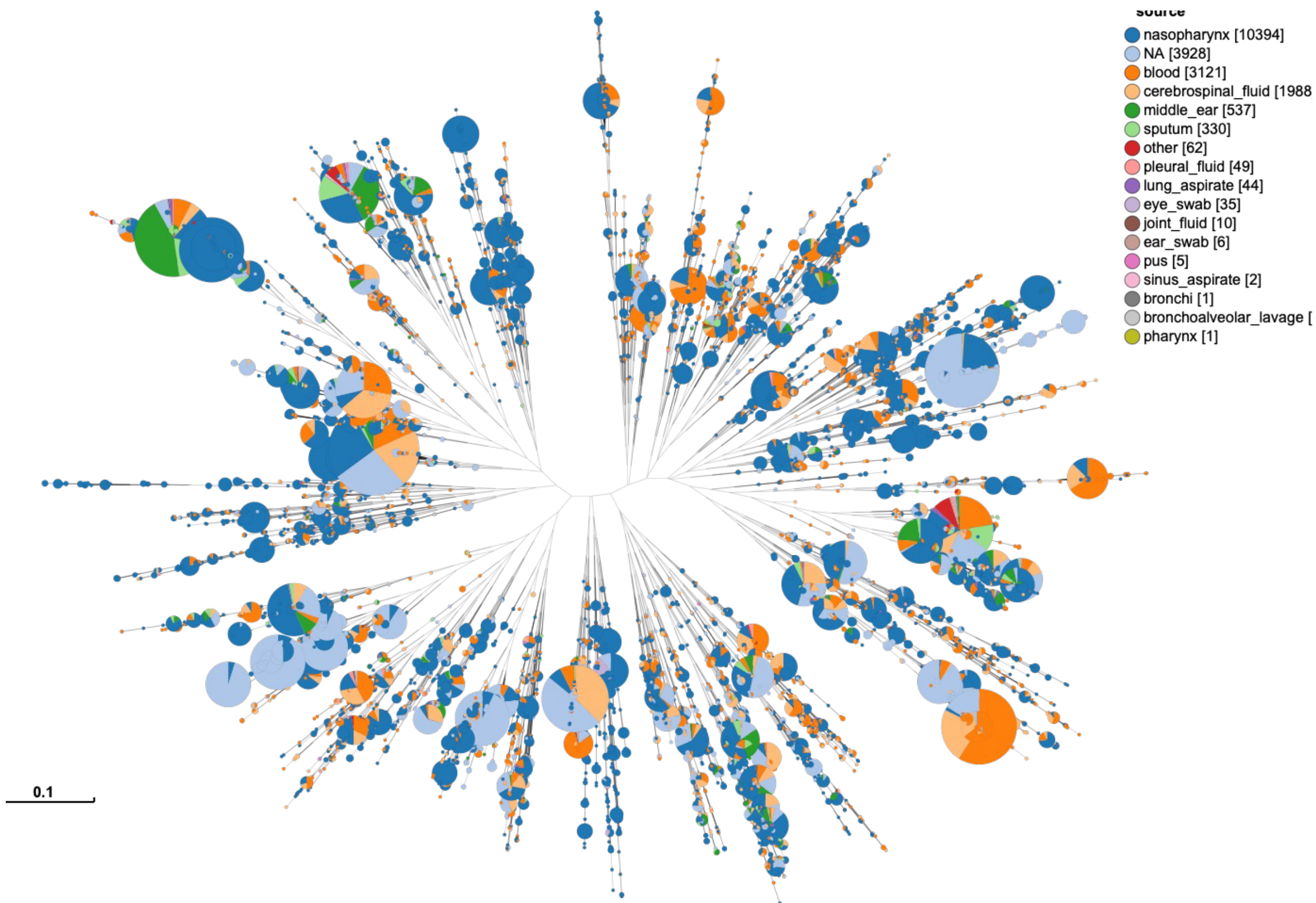


**Fig. S8. Minimum Spanning Tree (MST) Analysis of Pneumococcal Isolates Coloured by Anatomical Site of Isolation.** This figure depicts a minimum spanning tree (MST) analysis of pneumococcal serotypes categorised by their isolation site. Each node in the tree represents an individual genetic cluster, with colours indicating the specific body site from which the bacteria were collected, including the nasopharynx, blood, cerebrospinal fluid, middle ear, sputum, pleural fluid, lung, bronchi, sinus, and pharynx. The length of the branches between nodes reflects the genetic distance between serotypes, where shorter branches denote closely related isolates. This analysis highlights the genetic relationships between strains isolated from different anatomical sites and provides insights into the site-specific distribution and diversity of pneumococcal serotypes, which is important for including disease progression and transmission dynamics across various infection sites, towards the optimisation of ZPY-based formulation as a broad coverage vaccine.

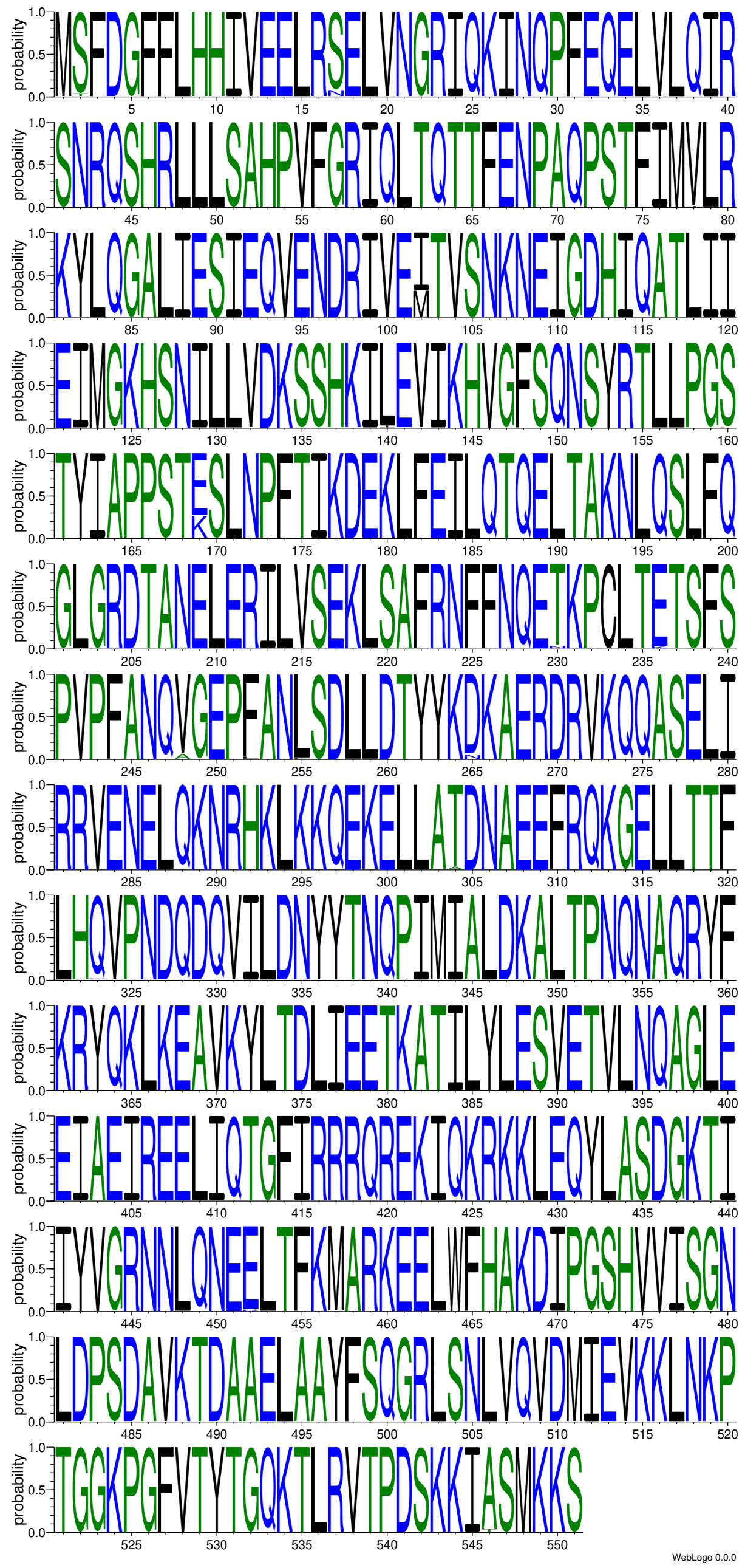

**Fig. S9. Amino Acid Multiple Sequence Alignment of PavA in a Quality filtered Dataset of Genomes.** This figure presents a multiple sequence alignment of the PavA gene across a quality filtered dataset of 11,304 pneumococcal genomes. The sequences are colour-coded based on the polarity of amino acid residues: black indicates non-polar residues, green represents polar uncharged residues, red denotes negatively charged residues, and purple indicates positively charged residues. The height of each letter within a column reflects the degree of conservation at that specific position, with taller letters indicating higher conservation across the genomes. Columns with two or more letters in the same position represent sites where multiple allelic variants exist, showing the presence of genetic diversity at that location within the population. This alignment highlights both conserved regions and areas of genetic variation in PavA, important for understanding its role in pneumococcal biology and potential as a vaccine antigen.

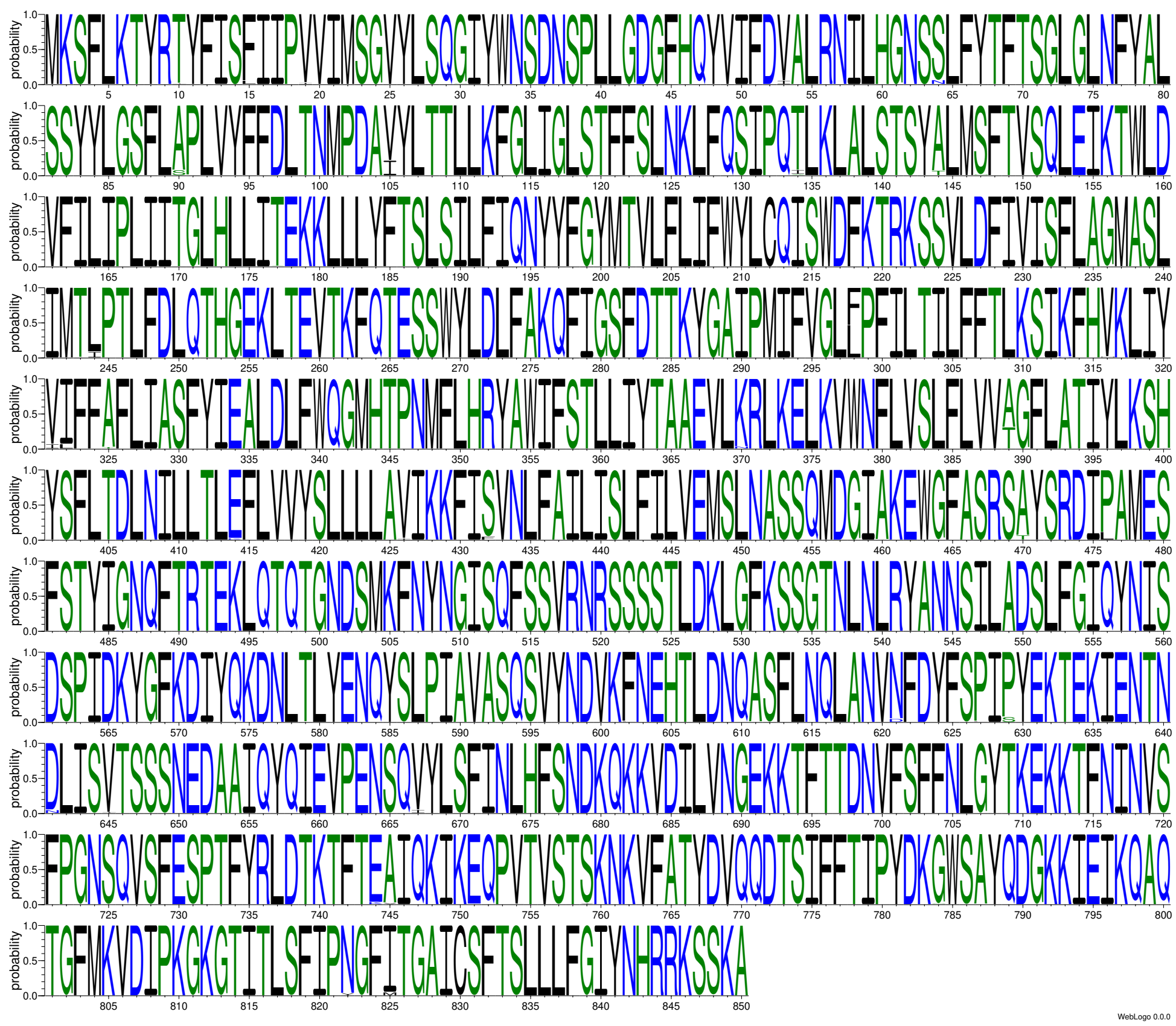

**Fig. S10. Amino Acid Multiple Sequence Alignment of YfhO-like in a Quality filtered Dataset Genomes.** This figure presents a multiple sequence alignment of the YfhO-like gene across a quality filtered dataset of 11,186 pneumococcal genomes. The sequences are colour-coded based on the polarity of amino acid residues: black indicates non-polar residues, green represents polar uncharged residues, red denotes negatively charged residues, and purple indicates positively charged residues. The height of each letter within a column reflects the degree of conservation at that specific position, with taller letters indicating higher conservation across the genomes. Columns with two or more letters in the same position represent sites where multiple allelic variants exist, showing the presence of genetic diversity at that location within the population. This alignment highlights both conserved regions and areas of genetic variation in YfhO-like, important for understanding its role in pneumococcal biology and potential as a vaccine antigen.

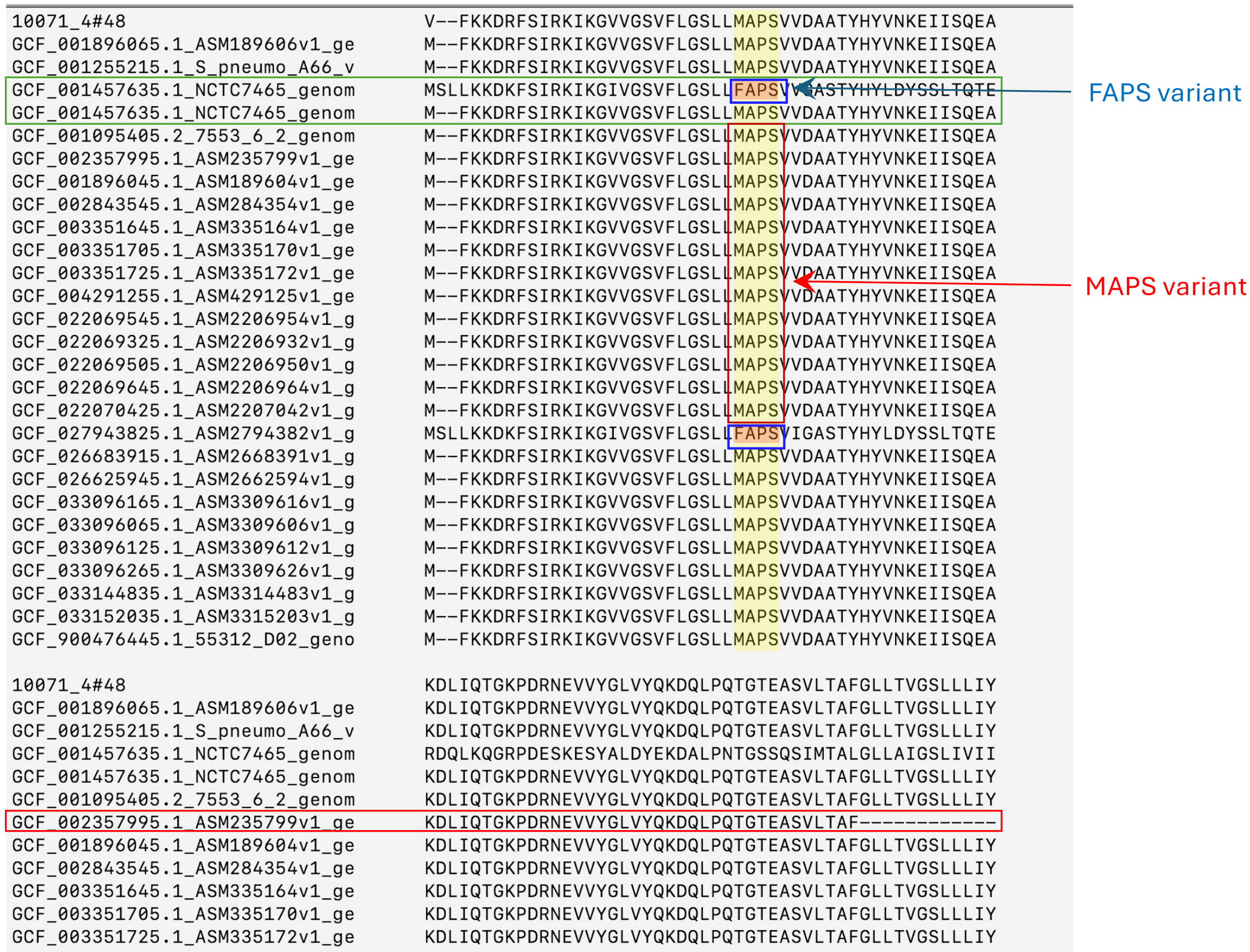


**Fig. S11. Multiple Sequence Alignment of the First 255 Amino Acids of ZmpB in a Representative Genomes Subset.** This figure illustrates the amino acid multiple sequence alignment of the first 255 residues of the ZmpB protein across a representative dataset of n=15,979 pneumococcal genomes. The alignment reveals that the initial 255 amino acids are highly conserved, followed by varying numbers of repeat domains in the central region. The C-terminal region also shows a reasonable level of conservation, though it appears to consist of two distinct types. Two distinct variants can indeed be visually identified within the alignment, referred to as MAPS and FAPS. All analysed strains exhibit the MAPS variant, while two strains additionally possess the FAPS variant. Notably, the AMS235799v1 strain and strain 55312 exclusively contain the MAPS variant, matching the sequence up to amino acid 86 almost identically. This analysis highlights both the conservation and diversity of ZmpB, which have implications for its role in pneumococcal virulence and vaccine development.

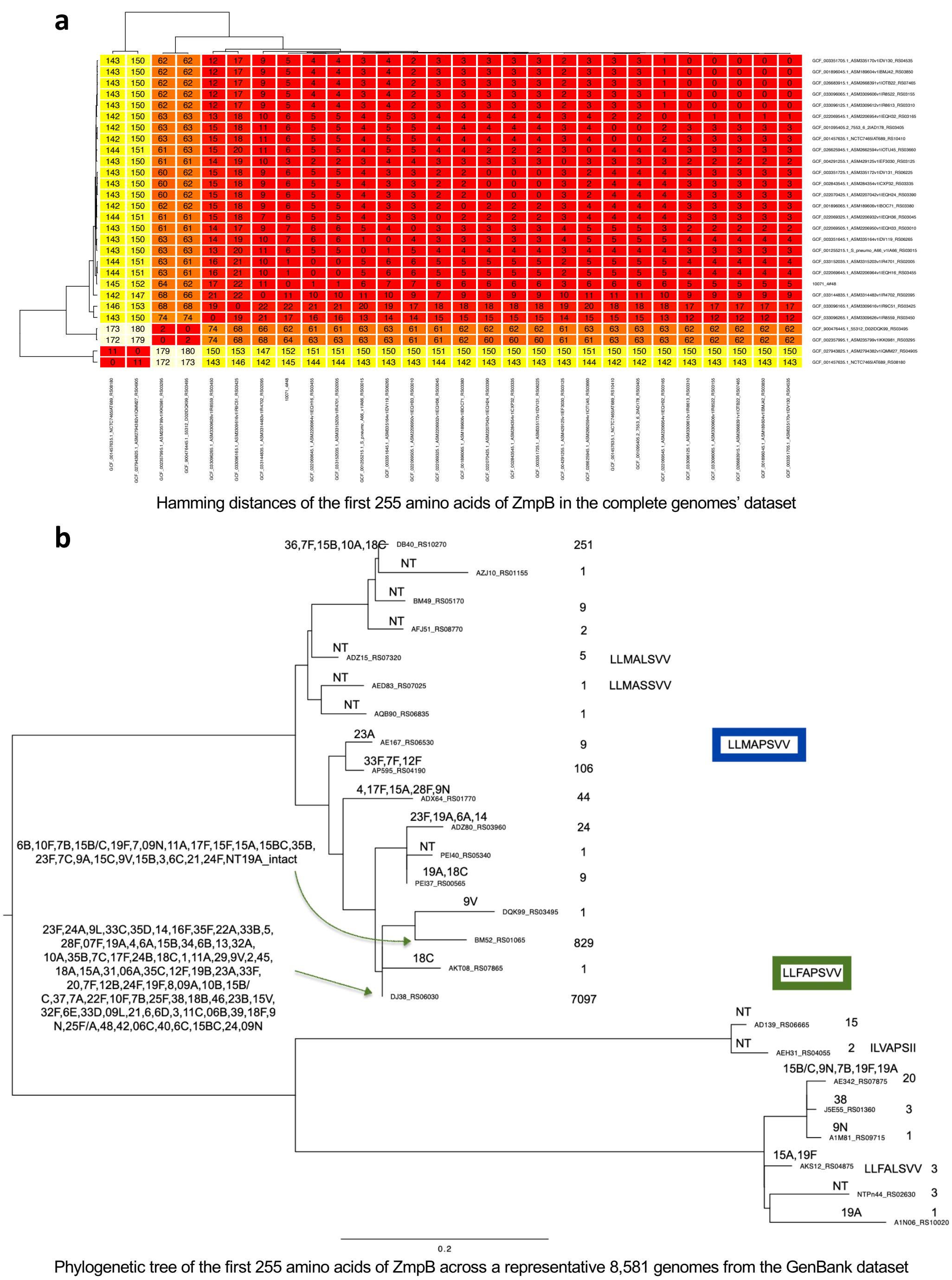


**Fig. S12. In-Depth Analysis of the Conserved 255 Amino Acids Region of ZmpB.** (a) The panel presents the Hamming distances calculated for the first 255 amino acids of the ZmpB protein across 15,979 genomes from the GenBank dataset. The Hamming distance metric provides insight into the genetic variability among different strains, highlighting conserved regions and identifying divergent sequences. (b) The panel displays the phylogenetic tree constructed from the first 255 amino acids of ZmpB across the entire set of genomes. The tree illustrates the evolutionary relationships among the strains based on the conserved region, allowing for the identification of clades and providing context for the diversity observed in the Hamming distance analysis. Together, these analyses contribute to a deeper understanding of the evolutionary dynamics and functional conservation of ZmpB, which may inform future research and vaccine development strategies against pneumococcal infections.

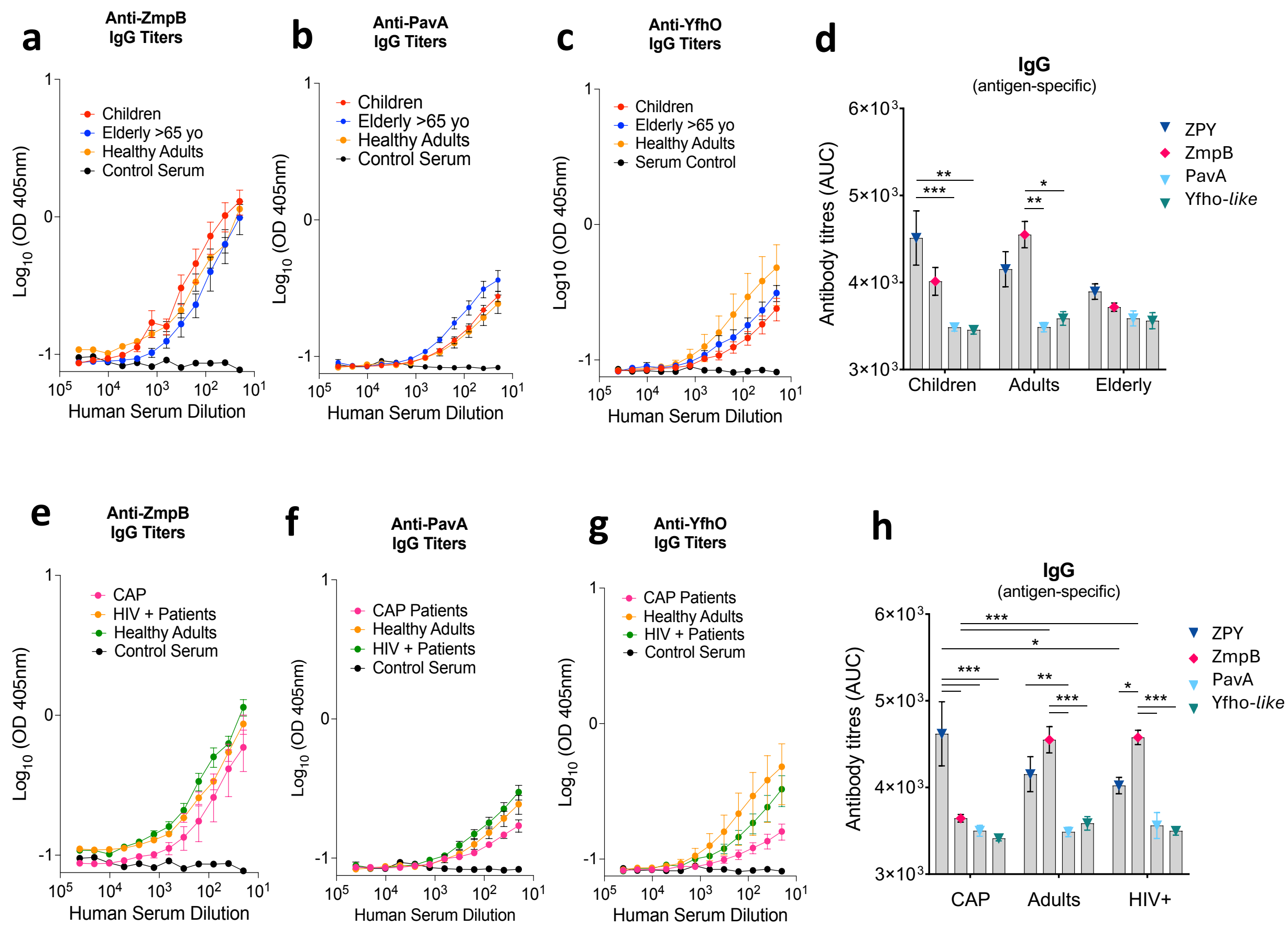


**Fig. S13. Serum IgG antibody titres to ZPY pneumococcal vaccine candidates (individual antigen measurements) in healthy and at-risk patient groups.** High-binding ELISA microplates were coated with individual ZmpB (a, e), PavA (b, f), or YfhO-like (c, g) antigens to assess specific IgG titres in the following subject groups: children aged 3 months to 7 years (red circles), healthy adults (orange circles), adults with community-acquired pneumonia (pink circles), HIV-positive adults without a history of pneumococcal infection (orange circles), and elderly patients (dark blue circles). Data represent the mean optical density (OD405nm) +/- SEM for each group. A total of 31 serum samples were analysed: n=13 children, n=5 healthy adults, n=5 adults with community-acquired pneumonia, n=5 HIV-positive adults without pneumococcal infection, and n=3 elderly patients. Optical density 450nm (OD450) values are presented as area under the curve in panels (i) and (j). Statistical significance was determined using Kruskall-Wallis with Dunn's post-test. Significance levels: *p < 0.05; **p < 0.01; ***p < 0.001.

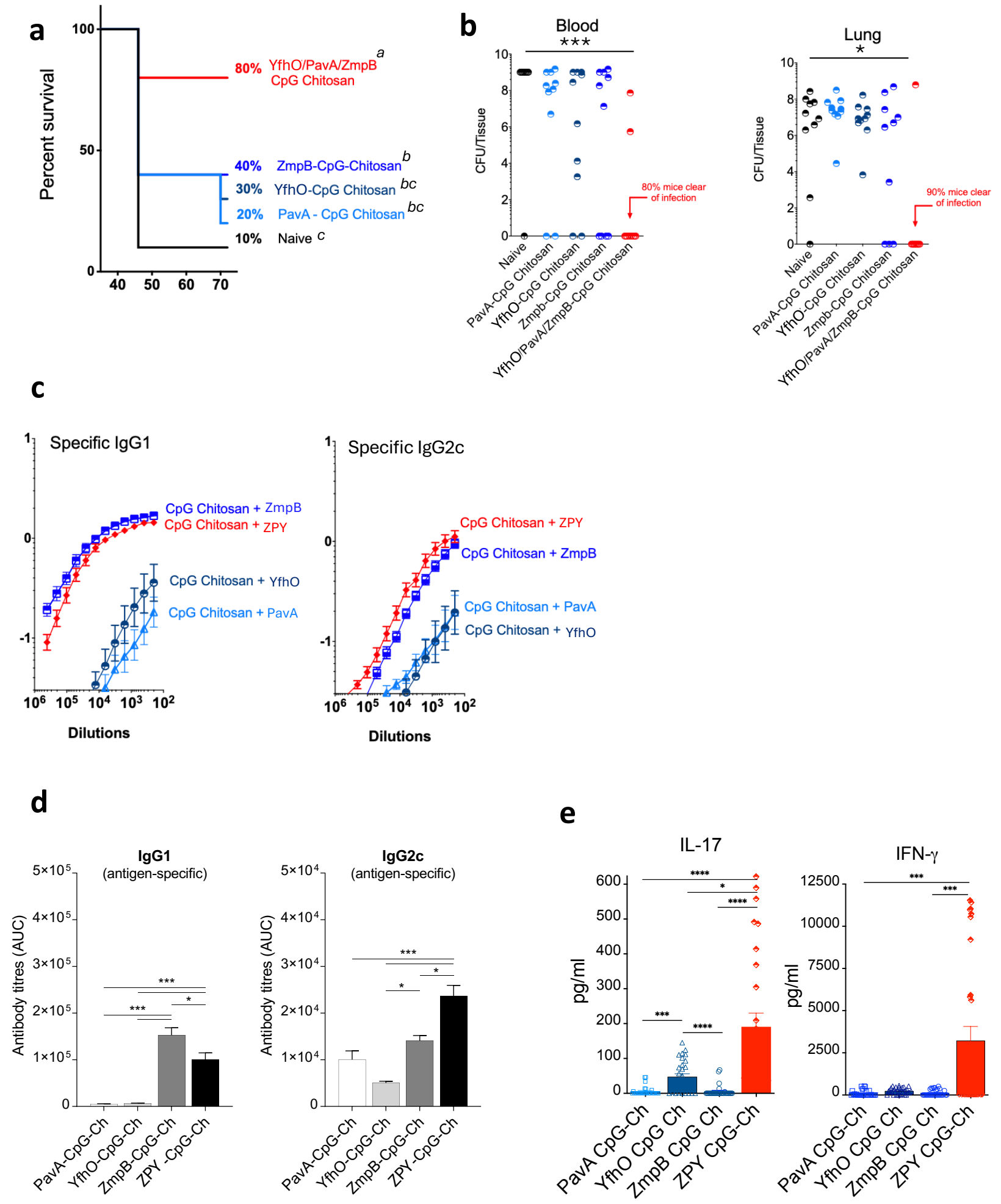


**Fig. S14. Vaccination with full ZPY-CpG-Ch Formulation Enhances Immunogenicity and Protective Efficacy Compared to Single Protein Adjuvanted CpG Formulations.** (a) Survival curves for adult mice (n=10 per group) vaccinated with various formulations: PBS (Vehicle), single antigen + CpG-Ch (1.0 ug of individual antigen formulated with CpG-Ch), or ZPY-CpG-Chitosan formulation, comprising PavA, YfhO-like, and ZmpB (1.0 ug) formulated with CpG-Ch. Mice were challenged intranasally with 5 x 10^5 CFU of a hypervirulent pneumococcal serotype 1 clinical isolate 35 days post-vaccination and were euthanized upon the onset of disease symptoms. (b) Viable counts (CFUs) of bacteria in the blood and lungs of immunised mice following challenge with pneumococci were assessed at the time of death. (c, d) Antibody dose-response analyses for single protein vs. mixed protein immunisation were conducted. Antigen-specific antibody levels (IgG1 and IgG2c) in the serum of vaccinated mice (n=10) were measured by ELISA. Results are expressed as mean +/- SEM, shown in OD units (optical density at 450 nm) (c) and area under the curve (AUC) (d). (e) Cytokine response analyses for single protein vs. mixed protein immunisation were conducted. Cytokines IFN-gamma and IL-17A cytokine concentrations were measured in the supernatant of mesenteric lymph nodes (MLN) collected from vaccinated mice (n=10) by ELISA using commercial immunoassays. Results are expressed as mean +/- SEM. The following statistical tests were employed: Survival analysis utilised the log-rank (Mantel-Cox) test, different letters indicate significant differences; bacterial loads (CFUs) and IgG titres (AUC) were analysed using the non-parametric Kruskal-Wallis test with Dunn's multiple comparisons. Significance levels: *$p < 0.05$; **$p < 0.01$; ***$p < 0.001$. Different letters indicate significant differences.

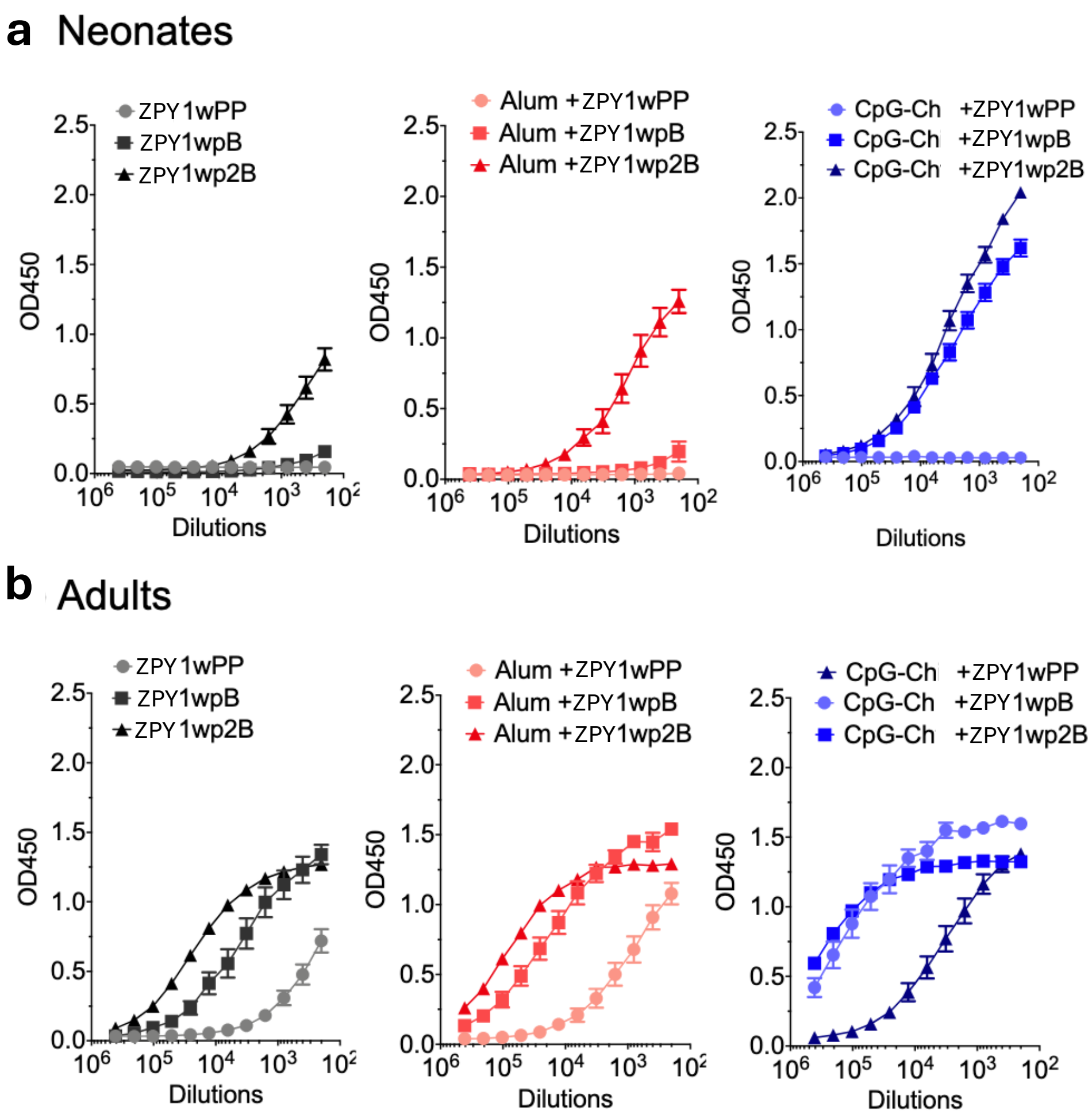


**Fig. S15. Optimisation of Prime-Boost Immunisation Regimen with ZPY-Adjuvanted Formulations.** Neonate (a) and adult (b) mice were immunised with ZP alone (comprising PavA, ZmpB, and YfhO-like in the vehicle), ZPY adsorbed to Alum, or ZPY adsorbed to CpG-Chitosan (CpG-Ch). Blood samples were collected via tail venipuncture at 1-week post-priming (black), 1-week post-booster 1 (red), and 1-week post-booster 2 (blue). Serum was then separated and analysed by ELISA for antigen-specific IgG (n=10 mice per group). The initial serum dilution was set at 1:200. Data are presented as mean +/- SEM (error bars). Abbreviations: 1wPP: sera collected at 1-week post-priming dose; 1wpB: sera collected at 1-week post-booster 1; 1wp2B: sera collected at 1-week post-booster 2.

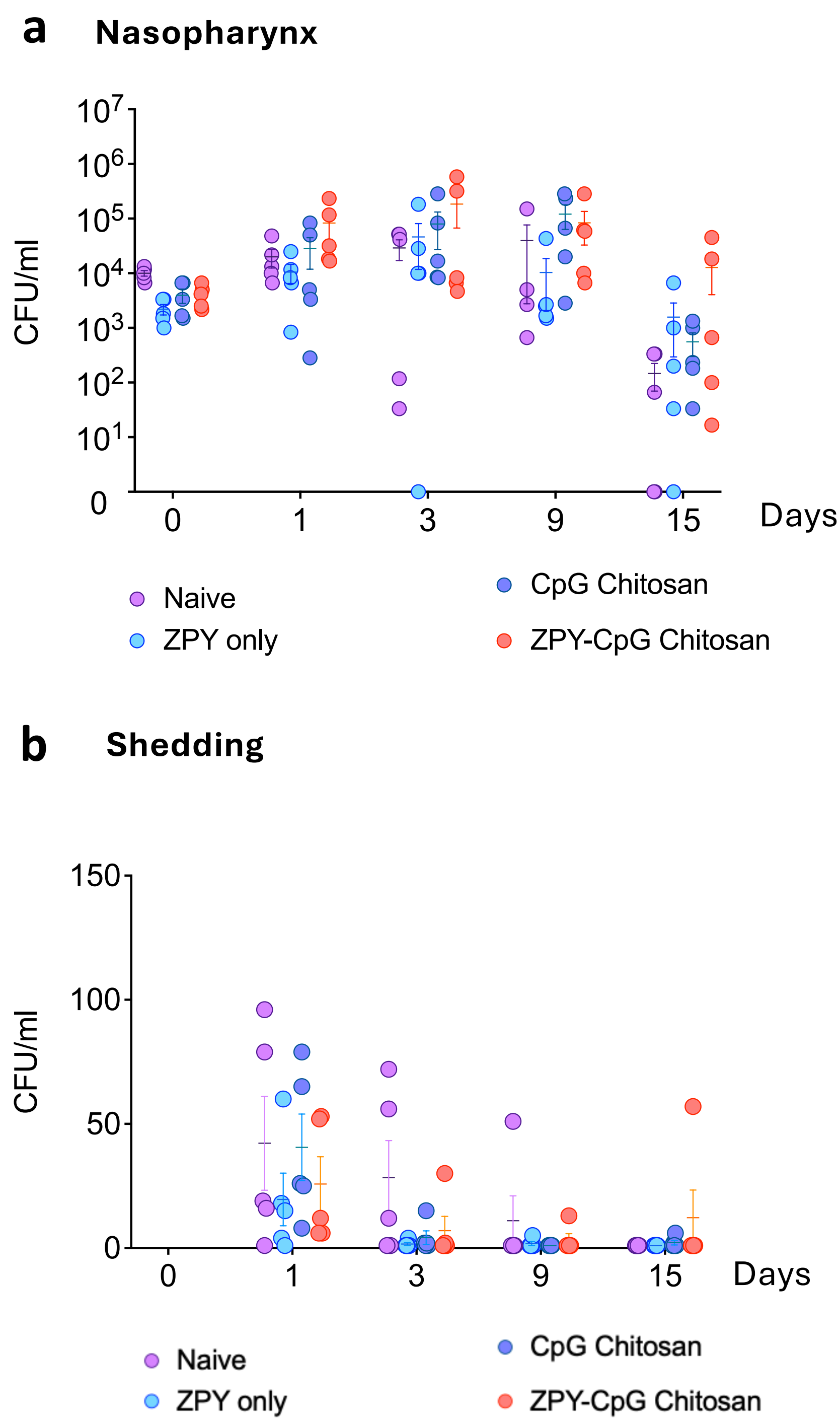


**Fig. S16. Impact of ZPY-CpG-Ch Immunisation on Subsequent Pneumococcal Nasopharyngeal Colonisation in Mice.** This study aimed to determine whether ZPY-CpG-Chitosan (ZPY-CpG-Ch) immunisation affects pneumococcal nasopharyngeal carriage in mice. Mice (n=5 per group) were immunised with either Vehicle (PBS), ZPY antigens (containing PavA, YfhO-like, and ZmpB in PBS), CpG-Chitosan (CpG-Ch) adjuvant alone, or ZPY adsorbed to CpG-Chitosan (ZPY-CpG-Ch). Two weeks after the second booster dose, mice were intranasally challenged with 10^5 CFU in 10 ul to establish nasopharyngeal colonisation. Nasopharyngeal tissue samples were collected at days 0, 1, 3, 9, and 15 post-challenge for colony-forming unit (CFU) counts, reported in (a) as CFU/ml. Additionally, on the same collection days, mouse noses were tapped onto agar plates to assess nasal shedding, as shown in (b). No statistically significant differences were observed between groups.

**Table S1. List of Bacterial Strains Used in this Study.**

| Genus | Species | Serotype | Strain / Source |
|---|---|---|---|
| Streptococcus | pneumoniae | 1 | Hyper-virulent African ST217. Malawi-Liverpool Wellcome Trust Centre, Malawi. |
| Streptococcus | pneumoniae | 8 | Strain ref no. 205521. Royal Liverpool University Hospital, Liverpool, UK. |
| Streptococcus | pneumoniae | 11 | BHN-906_11A. Karolinska Institute, Stockholm, Sweden. |
| Streptococcus | pneumoniae | 33F | Strain ref no. 193466. Royal Liverpool University Hospital, Liverpool, UK. |

**Table S2. Mouse Pneumonia Model Pain Score.**

| Status | Score | Description |
|---|---|---|
| Normal | 0 | Mouse is moving around cage normally and has a normal coat |
| Hunched + | 1 | Mouse is slightly arched over and walks on tip toes |
| Hunched ++ | 2 | Mouse is very arched over and walks on tip toes |
| Starry / piloerect + | 2.5 | Mouse fur is upright around the neck area |
| Starry / piloerect ++ | 3.0 | Mouse coat is upright all over the mouse |
| Lethargic + | 3.5 | Mouse is slower moving around the cage |
| Lethargic ++ | 4.0 | Mouse is not moving around the cage unless encouraged |
| Moribund | 4.5 | Mouse has stopped moving, coat is starry and upright, breathing is very laboured |

*Adapted from: Morton DB, Griffiths PH. Guidelines on the recognition of pain, distress and discomfort in experimental animals and hypothesis for assessment. Vet Rec. 1985 Apr 20;116(16):431-6. doi: 10.1136/vr.116.16.431.*

**Table S3. List of Flow Cytometry Antibodies Used in this Study.**

| Antibody | Reactivity | Source | Identifier |
|---|---|---|---|
| FITC anti-mouse CD3 | Mouse | Biolegend | Clone 17A2, Cat# 100204 |
| APC-Cy7 anti-mouse CD4 | Mouse | Biolegend | Clone GK1.5, Cat# 100414 |
| APC anti-mouse CD4 | Mouse | Biolegend | Clone GK1.5, Cat# 100412 |
| PE anti-mouse IL-17A | Mouse | Biolegend | Clone TC11-18H10.1, Cat# 506904 |
| APC anti-mouse IL-4 | Mouse | Biolegend | Clone 11B1, Cat# 504106 |
| Brilliant Violet 421 anti-mouse IFN-gamma | Mouse | Biolegend | Clone XMG1.2, Cat# 505830 |
| Pacific Blue anti-mouse CD62L | Mouse | Biolegend | Clone MEL-14, Cat# 104424 |
| PE-Cy7 anti-mouse/Human CD44 | Mouse / Human | Biolegend | Clone IM7, Cat# 103030 |
